# Initiation is recognized as a fundamental early phase of integrated knowledge translation (IKT): qualitative interviews with researchers and research users in IKT partnerships

**DOI:** 10.1186/s12913-019-4573-4

**Published:** 2019-10-30

**Authors:** Maria Maddalena Zych, Whitney B. Berta, Anna R. Gagliardi

**Affiliations:** 1 Institute of Health Policy, Management and Evaluation, University of Toronto, 155 College Street, Suite 425, Toronto, Ontario M5T 3M6 Canada; 2 Toronto General Hospital Research Institute, University Health Network, 200 Elizabeth Street, 13EN-228, Toronto, Ontario M5G 2C4 Canada

**Keywords:** Integrated knowledge translation, Qualitative research, Partnership initiation

## Abstract

**Background:**

Health care researcher-research user partnerships, referred to as integrated knowledge translation (IKT), have been adopted on an international basis, and are an effective means of co-generating and implementing evidence into policy and practice. Prior research suggests that an initiation period is essential for establishing functional partnerships. To characterize IKT initiation and describe determinants of IKT initiation success, this study explored IKT initiation processes, enablers, and barriers among researchers and research users involved in IKT partnerships.

**Methods:**

A descriptive qualitative approach was used compliant with COREQ standards. Canadian researchers and research users in research collaborations were identified on publicly-available directories and web sites, and referred by those interviewed. They were asked to describe how partnerships were initiated, influencing factors, the length of initiation, and interventions needed to support initiation. Sampling was concurrent with data collection and analysis to achieve thematic saturation. Data were analyzed using constant comparative technique by all members of the research team.

**Results:**

In total, 22 individuals from 6 provinces were interviewed (9 researchers, 11 research users, 2 connectors). They confirmed that IKT initiation is a distinct early phase of partnerships. The period ranged from 6 months to 2 years for 75.0% of participants in pre-existing partnerships, to 6 years for newly-formed partnerships. High-level themes were: Newly identifying and securing partners is an intensive process; Processes and activities take place over a protracted period through multiple interactions; Identifying and engaging committed partners is reliant on funding; and Partnership building is challenged by maintaining continuity and enthusiasm. Participants underscored the need for an IKT partner matching forum, IKT initiation toolkit, and funding for non-research activities required during IKT initiation to establish functional researcher-research user partnerships. Themes were largely similar regardless of participant years of experience with IKT or being involved in a new versus pre-existing partnership.

**Conclusions:**

IKT initiation is a recognized and important early phase of IKT that establishes functional partnerships, and once established, ongoing partnership for subsequent projects is likely. Further research is needed to develop and evaluate approaches recommended by participants for stimulating IKT initiation.

## Background

Knowledge or evidence can be implemented once generated using a variety of strategies or interventions aimed at individuals, organizations or health systems [1]. Alternatively, it is now well-recognized that partnerships between researchers and research users who co-generate knowledge, a process often referred to as integrated knowledge translation (IKT), are an effective means of enabling the uptake of research evidence to optimize healthcare planning, delivery, and outcomes [2–4]. IKT can empower research users that were traditionally passive consumers of knowledge to have a say in its creation or direction [5, 6], create capacity to make research evidence context-sensitive to local health care needs [7], and prime knowledge users, who may be involved in various steps of the research process, to apply that knowledge [8]. Research users include clinicians, managers, patients, family members or caregivers, health system leaders or policy-makers, and other stakeholders who may use the research [9]. Researchers include individuals who are employed in various settings such as a research institute or university where they apply research methods to produce knowledge. Research users can be involved in any step of the research process; for example, shaping of the research question, choosing methodology, developing data collection instruments, collecting data, analyzing and interpreting the findings, and helping to disseminate results [3, 9, 10]. Hence, in the context of implementation science, IKT represents an important strategy that promotes and supports the translation of research into policy and practice.

Large scale examples include the Collaborations for Leadership in Applied Health Research and Care (CLAHRCs) in the United Kingdom (UK) [11] or the Academic Collaborative Centres (ACCs) in the Netherlands [12]. In both cases, considerable resources were invested at a national level in establishing the capacity to support partnerships between researchers and research users, leading to increased IKT activity, and use of evidence to inform policy and practice [7, 11]. Research based on these and other partnerships revealed numerous factors influence IKT and its impact; they include establishing a physical space for communication and collaboration, preferably at close geographical proximity [2, 13], setting roles, goals and responsibilities for team members [2, 14–16], setting leadership for projects [2, 7], and funding for grants and incentives [7, 16]. When absent in either researchers or research users, the following factors function as barriers of IKT: positive attitude towards IKT [2, 17], knowledge about IKT [18, 19], funding or other incentives to take part in IKT [17], time to commit [2, 20], sense of belonging to a research team [21], and trust and respect between partners [13, 20].

Prior IKT research suggests that there may be an initiation phase preceding active research collaboration. For example, Gagliardi et al. performed a scoping review of 13 IKT studies published from 2005 to 2014 [2]. While all IKT initiatives were in place for at least 2 years, and in some cases up to 8 years, the numerous barriers of IKT identified were largely barriers relevant to the formation of partnerships such as identifying willing partners, and establishing goals, roles and responsibilities [2]. In 2011, Kothari et al. identified indicators for IKT through interviews with 16 researchers and research users involved in eight IKT partnerships, and then validated the indicators during two-hours focus groups with researchers and researcher users [22]. Indicators of IKT specific to the first 2 years of partnership included negotiation of roles, developing a team mentality, ensuring clear leadership, and engaging team members [22]. Research on the CLAHRCs also contributed to the conceptualization of IKT initiation. A longitudinal realist evaluation was conducted over the period of 2009 to 2014 on three CLAHRC sites to explain how and why the programs were successful [11]. The authors grouped the process of IKT into five phases. One of the phases focused on initiation, and was described as a time when cognitive, conceptual and physical relationships were being developed [11]. An important aspect of this stage was leadership for creating change, and the ability to delegate leadership to people who were most closely involved with partnership projects [11]. Activities associated with IKT initiation may include establishing virtual and physical convenient communication spaces and channels that will work through the duration of the project [2, 23, 24]; defining team mentality by clarifying or establishing visions, mission, goals, terms of reference, rules, regulations, policies, priorities and project timelines, as well sharing continuous evaluation or progress updates with the stakeholders [2, 22, 24]; planning to negotiate roles at different stages, identifying member skills and delegating work [2, 22]; and ensuring clear leadership and the sustained engagement of team members [11, 22, 24, 25].

Clearly IKT initiation is crucial to the subsequent functioning and beneficial outcomes of IKT partnerships, but it has never been thoroughly examined to characterize IKT initiation and describe factors that contribute to IKT initiation success. The purpose of this study was to explore IKT initiation processes, enablers, and barriers among health care researchers and research users involved in IKT partnerships.

## Methods

### Approach

This exploratory study was based on qualitative interviews with researchers and researcher users to thoroughly understand the experiences of being involved in IKT partnerships [26]. Interviews were conducted and analyzed using a basic descriptive qualitative approach, which aims to describe experiences by staying data-near or as close to the accounts conveyed by participants as possible [27], rather than employing or generating theory as in other qualitative research traditions [28]. We complied with the Consolidated Criteria for Reporting Qualitative research (COREQ) [29]. Research ethics approval was granted by the University of Toronto’s Office of Research Ethics.

### Sampling and recruitment

We used purposive sampling to recruit participants currently or recently involved in IKT partnerships who varied in characteristics that could influence their views or experiences including type of participant (researcher, researcher user), years of experience in IKT, having previous relationships versus starting new relationships for the partnership, and geographically dispersed across Canada [26]. Individuals were identified through a national IKT-based research network that lists the names and contact information of researchers and research users on their website [30], and by searching the Canadian Institute for Health Research (CIHR) Funded Research Database for recipients of Partnerships for Health System Improvement (PHSI) research grants, which required that researchers partner with research users. We sampled recipients of PHSI grants in the 3 years prior to our study (2014 to 2017). Snowball sampling, meaning referral to additional researchers or research users by interviewed participants, was also employed. Individuals were contacted by email with an invitation to participate. Participants were asked to sign and return an informed consent form prior to scheduling an interview. The research team had no prior relationship with the participants.

### Data collection

MZ, with prior training in qualitative research and mentored by ARG, conducted semi-structured telephone interviews. Questions were derived from previous readings of IKT literature, and focused on processes, enablers and barriers of IKT initiation. Participants were asked six questions listed in Table 1 below:

**Table 1:** Interview questions

Briefly describe the objective of the research that you were involved in and your role;	
How was partnership initiated?	
What activities or types of interaction took place during initiation?	
What factors enabled partnership initiation?	
What factors were barriers of partnership initiation?	
What strategies or interventions or tools would support partnership initiation?	

Depending on responses, participants were prompted to expand on their experiences. Interviews conducted between February 1 and May 8, 2018, were audio-recorded and transcribed verbatim. The duration of interviews ranged from 22:20 to 104:36 min. Interviews were conducted until thematic saturation occurred [31], as determined by MZ during prospective transcript analysis, and review and discussion by the research team (ARG, WBB).

### Data analysis

Data were tabulated and organized using typical office software (MS Word and Excel). Transcripts were not returned to participants for review. Unique themes were identified using constant comparison [32]. One transcript was independently analyzed by ARG, MZ and WBB to generate a preliminary coding scheme comprised of themes and exemplar quotes. To elaborate the coding scheme, two additional transcripts were analyzed by MZ and ARG independently, then discussed by the research team. MZ analyzed the remaining transcripts and compiled themes and exemplar quotes, which were reviewed by ARG and WBB on six occasions. Data were tabulated to identify similarities and differences in themes by participants’ characteristics such as role in the project (researcher, research user), years of experience in IKT partnerships, and pre-existing or new IKT relationship.

## Results

### Participants

A total of 63 individuals were invited to participate in the interviews. Two declined to participate because they felt they did not meet eligibility criteria, one declined due to lack of time. Thirty-eight individuals did not respond to the invitation. Twenty-two individuals were interviewed, 18 females (81.8%) and 4 males (18.2%). These included 9 (40.9%) researchers, 11 (50.0%) research users, and 2 (9.1%) participants who self-identified as connectors of researchers and research users. Participants self-reported that they had 5 to 10 years (4, 18.2%) or more than 10 years of IKT experience (18, 81.8%). Participants were based in several different Canadian provinces: Ontario (7, 31.8%), Manitoba (5, 22.7%), Alberta (4, 18.2%), British Columbia (4, 18.2%), and 1 (4.5%) each from Quebec and Nova Scotia. Among 20 researchers or research users, 15 (75.0%) represented pre-existing partnerships. The length of time for IKT initiation was said to range from 6 months to 6 years. Even for pre-existing partnerships, the IKT initiation period for a new research project ranged from 6 months to 2 years. A summary of participant characteristics is provided in Table 2. Data including themes and quotes are available in Additional file 1.

**Table 2:** Participant characteristics

ID	Job Title	Interview date	Participant type	IKT experience (year)	Province	IKT initiation period (years or months)	New or pre-existing partnership	Interview duration (minutes)
C01	Executive Director	6-Feb-18	Connector	Over 10	Alberta	Six months to year	Pre-existing	46:52
C02	Chief Executive Officer	3-Apr-18	Connector	Over 10	Manitoba	Not reported	Pre-existing	33:38
R01	Clinician Scientist and Emergency Physician	1-Feb-18	Researcher	5 to 10	Ontario	2007–2013 (6 years)	New	58:48
R02	Microbiologist	12-Feb-18	Researcher	Over 10	Ontario	6 months	Pre-existing	33:51
R03	Health Services Researcher	15-Feb-18	Researcher	5 to 10	British Columbia	2016 to about one month ago (26 months)	Pre-existing	30:14
R04	Assistant Professor	16-Feb-18	Researcher	Over 10	Manitoba	October 2013 to June 2015 (20 months)	New	32:23
R05	Associate Professor and Associate Director	27-Feb-18	Researcher	Over 10	British Columbia	2011 to 2013 (24 months); grant awarded in 2013	Pre-existing	104:46
R06	Assistant Professor	13-Mar-18	Researcher	Over 10	Ontario	Over a year	New	36:20
R07	Associate Professor and Division Head	14-Mar-18	Researcher	Over 10	Alberta	2011 to 2013 (24 months)	New	38:50
R09	Assistant Professor and Scientist	22-Mar-18	Researcher	Over 10	Halifax	one year	Pre-existing	32:17
R10	Associate Professor	28-Mar-18	Researcher	Over 10	Quebec	6 to 8 months	Pre-existing	32:56
U01	Executive Director	13-Feb-19	Research User	Over 10 years	Ontario	Not reported	Pre-existing	32:40
U02	Director	15-Feb-18	Research User	5 to 10 years	Manitoba	2 years	New	28:53
U03	Program Director	8-Mar-18	Research User	Over 10 years	Manitoba	2012 to 2016 (4 years)	Some new some pre-existing	22:20
U04	Regional Director	15-Mar-18	Research User	Over 10 years	Manitoba	Six months to one year	Pre-existing	42:04
U05	Director	29-Mar-18	Research User	Over 10 years	Alberta	Not reported	Pre-existing	29:54
U06	Director	5-Apr-18	Research User	Over 10 years	British Columbia	Not reported	Pre-existing	36:35
U07	President and Chief Executive Officer	20-Apr-18	Research User	Over 10 years	British Columbia	Not reported	Pre-existing	29:45
U08	Assistant Professor and Director	30-Apr-18	Research User	Over 10 years	Ontario	Not reported	Pre-existing	29:26
U09	Senior Director, Strategic Partnerships and Priorities	01-May-18	Research User	Over 10 years	Alberta	Not reported	Pre-existing	32:54
U10	Director, Person Centre Care	04-May-18	Research User	Over 10 years	Ontario	Not reported	Pre-existing	35:50
U11	Director, Patient, Caregiver and Public Engagement	08-May-18	Research User	Over 10 years	Ontario	Not reported	Pre-existing	23:22

### Summary of key themes

Themes that characterize IKT initiation are summarized in Table 3 along with related activities and are described here with exemplar quotes: Newly identifying and securing partners is an intensive process; Processes and activities take place over a protracted period through multiple interactions; Identifying and engaging committed partners is reliant on funding; Partnership building is challenged by maintaining continuity and enthusiasm; and a Partner matching forum, toolkit and funding for non-research activities are needed to foster IKT initiation. Themes were largely similar regardless of participant years of experience with IKT or being involved in a new versus pre-existing partnership.

**Table 3:** IKT initiation themes and associated activities

Theme	Activities
Newly identifying and securing partners is an intensive process	• Identify stakeholders • Conduct research together as an extension of the partnership • Contact individuals with dual role of researcher and research user • Convince and get partner buy-in on research project • Form partnership early and ensure collaboration lasts throughout
Processes and activities take place over a protracted period through multiple interactions	• Clarify roles, responsibilities, scope of project, research question by meetings, committees, agreements • Align idea with research user organization’s goals; educate each other on how to align goals • Provide opportunities for communication and input
Identifying and engaging committed partners is reliant on funding	People and Roles: • Build a network well in advance and have organizational capacity to maintain network • Use research project as incentive for research users to stay up to date and researcher to obtain tenure • Build and maintain a relationship with connectors, boundary spanners, or mentors • Leadership role • Early partnership formation and collaboration from the outset of a research initiative
	Building commitment: • Shared goals amongst the researchers and the research users themselves, or their organizations • Shared interest, synergy and passion for the subject matter • Trust and commitment achieved by being responsive, respectful of time, planning face-to-face meetings and maintaining credibility • Time to commit to partnership and creating time-saving methods for busy research users to take part in projects • Build a sense of ownership by making researcher users feel valued, respected and have a sense of ownership over research outputs • Develop a shared language and culture
	Funding • Enable initiation by supporting identification of partners • Enable travel for face-to-face meetings at beginning of partnerships • Establish commitment and launch planning activities
Partnership building challenged by maintaining continuity and enthusiasm	• Maintaining continuity during IKT initiation due to high turnover of individuals • Over-reliance on one person representing a research user group • Difficult to maintain enthusiasm throughout the first few meetings during the initiation phase • Engagement was further compromised by competing priorities among research users • The amount and extent of administrative paperwork at the beginning of partnerships also influenced enthusiasm • Personality of the researcher or research • Lack of understanding of the research cycle or research culture • Misaligned goals, roles and expectations. • Geographical distance between the partners
Partner matching forum, toolkit and funding needed to foster IKT initiation	• A shared forum or repository where researchers could identify potential researcher user partners who shared matching interests and goals • An IKT initiation toolkit, guide or checklist described as ‘how to start IKT partnership information’ • Funding for non-research IKT initiation activities such as travel and meetings

### Newly identifying and securing partners is an intensive process

When asked who initiated the partnerships, 4 said that it was researchers (18.2%), 7 said it was research users (31.8%), and in 5 (22.7%) instances the partnership arose or evolved as an extension of a previous partnership. When asked how the partnership was initiated, 14 (63.6%) said that they contacted individuals known from pre-existing relationships to ask if they would either participate, support, or endorse the partnership, or refer them to another potential participant. Some participants mentioned more structured and intensive approaches for identifying and securing partners, such as conducting environmental scans, analyzing pilot data or conducting capacity building workshops on KT topics to attract interested parties. Many researchers described having to convince potential partners by explaining why the product or service would be beneficial to the research users’ everyday work, often requiring multiple, sequential communications or even travelling to the prospective partner to deliver a presentation.

*I needed to get buy-in and approval from them and convince them that they would use knowledge from the project and the organization would benefit from that (R01).*

*]Finding existing meetings to bring it [idea] forward and then you know at the end of the day the biggest argument is but my patients are special or I’m special. And at the end of the day we said everybody’s special, help us determine what we mean by special and we got buy-in (U04).*

### Processes and activities take place over a protracted period through multiple interactions

Administrative and governance activities at the beginning of IKT partnerships included organizing meetings, establishing committees, or creating project agreements that were used to clarify roles and responsibilities (R01, R03, R04, R05, R07, U03, U04, U06, U07, U10, C02). Ten participants said it was not the type of activity that was important, but the ability to demonstrate the alignment of the idea with the research user organization’s goals and priorities (R01, R06, R07, U01, U04, U05, U06, U08, U10, C01). Some researchers said they assumed the role of educator to help research users understand and align the idea to the priorities of their organization (U01).

*If a researcher came and said, hey let’s set some priorities up, I might say, you need to start with some of the key documents like in our case there’s [Provincial Health Priority Document]. You want to see what’s important to me, you go look at those documents and then come back and talk. So that’s part of, I find that part of my role is just educating researchers who want to make an impact…how do they engage in that conversation upon initiation because you might say, I’m really interested in some topic that isn’t even on the radar and you don’t line up to the you know the platform commitments or the budget commitments (U01).*

Research activities said to fall within the IKT initiation period included joint development of research funding applications or writing letters of support for funding applications (R01, R09, U06). All processes and activities took place over a protracted period of time through multiple interactions and communications via teleconference, email, special events, or workshops (R02, R03, R04, U08, U09, U10, C01).

### Identifying and engaging committed partners is reliant on funding

Enablers for identifying and engaging partners were organized in the categories of: People and Roles, Building Commitment, and Funding.

#### People and roles

Building a network from within which to support IKT initiation by identifying partners was considered important. This was expressed as having a pre-defined network of interested individuals or organizational capacity to build and support that network (R02, R03, R04, R05, R06, U01, U08, C01, C02). With respect to identifying partners, a minor enabler was related to recruitment, and included using the research project as an incentive, including presenting the partnership as a way for researcher users to stay up to date or for researchers to obtain tenure (R06, U08). Similar to the enabler of having a pre-defined network, another enabler mentioned by 11 participants was building or maintaining a relationship with individuals who act as connectors, boundary spanners or mentors between researchers and research users (R01, R03, R04, R05, R06, R07, R08, U01, U07, U08, C02). Taking a leadership role in initiating the partnership was an enabler that emerged in the responses from researchers, research users and connectors (R01, U07, U08, C01, C02). Early partnership formation and collaboration from the outset of a research initiative (R05, R06, R09, U01, U04, U06, U07, U09, C01, C02) was a common enabler that arose in 10 interviews.

*I think you really, really have to do a good job at pre-initiation as well as when you actually initiate the research (R05)*

*It would be nice if some people first start thinking about these applications…what we tend to get is like a, you know, a one, maybe two-page kind of summary of what the application is gonna look like. But you know it would have been helpful if we’d been brought in sometimes even earlier…it would have really helped to set us up and understand much clearer what they were working for and what they were doing. [When] there are opportunities to bring us in earlier, I think that would be really helpful (U06)*

*… to try and establish those kinds of relationships early on does make for successful approach… (C02)*

#### Building commitment

Another enabler that arose from the interviews was the idea of shared goals amongst the researchers and the research users themselves, or their organizations (R01, R06, R07, U01, U03, C01, C02). This was also described as having a shared interest, synergy and passion for the subject matter (R07, R09, U04, U05, U011). Participants emphasized trust and commitment, achieved by being responsive, respectful of time, planning face-to-face meetings, and maintaining credibility (R01, R05, R06, R07, U04, U07, U10, U11, C02). Other important enablers were time to commit to the partnership and creating time-saving methods for busy research users to take part in the projects (R02, R04, R05, U04, U08, U10, U11), building a sense of ownership, which included making research users feel valued, respected, and have a sense of ownership over the research outputs (R02, R04, R05, R06, R07, R09, U04, U06, U07, U08, U09, U10, U11, C02), and developing a shared language and culture (R04, R05, R07, R10, U07).

#### Funding

Funding was commonly reported as an essential element that would enable IKT initiation by supporting the identification of partners, (R01, U05, U06, U07, U08, C01, C02), including travel at the beginning to meet partners face-to-face (R01, R02, R05, U01, C02), and interaction among partners to establish commitment and launch planning activities.

#### Partnership building challenged by maintaining continuity and enthusiasm

Participants said that maintaining continuity during IKT initiation due to high turnover of individuals in research user organizations was a challenge (R01, R04, R06, R09), and also due to over-reliance on one person representing a research user group (R01, R04, U08). Some participants said that it was difficult to maintain enthusiasm throughout the first few meetings during the initiation phase (R01, R04, R05, U08). Engagement was further compromised by competing priorities among research users (R01, R02, R03, R05, R06, U01, U03, U04, U05, U08, C02). The amount and extent of administrative paperwork at the beginning of partnerships also influenced enthusiasm (R02, R10, U08).

*We’ve created these layers of necessary agreements that are now really onerous to maintain. I had in one of the great glories of life, I have an appointment at both [Hospital] and the [Larger Hospital Network]; I have data-sharing agreements with myself” (R02)*

Additional common barriers that influenced continuity and/or enthusiasm included the personality of the researcher or research (R01, R05, U01, U07, U08, U09, U10), lack of understanding of the research cycle or research culture (R01, R06, R10, U01, U02, U07), and misaligned goals, roles and expectations sometimes resulting from applying for grants without clearly defining the partnership (R05, R06, R09, R10, U03, U05, U06, U08, U09, U10). A minor barrier was geographical distance between the partners (R07, U07).

### Partner matching forum, toolkit and funding needed to foster IKT initiation

When asked about strategies, tools or interventions needed to facilitate IKT initiation, participants recommended a shared forum or repository where researchers could identify potential researcher user partners who shared matching interests and goals (R01, R07); an IKT initiation toolkit, guide or checklist described as ‘how to start IKT partnership information’ (R01, R06, R07, R10, U0 2, U04, U06, U07, U09, C01); and funding for non-research IKT initiation activities such as travel and meetings (R07, U02).

*…Well you know it would be great if there was some sort of clear document that you have that you would describe what your role is as a researcher and not just assume that it’s understood (R06)*

*Something that would be pretty amazing would be to have somebody write a document that says, so you think that you need to do research in any given area that you worked in. Here’s some of the questions that you need to ask, right? As opposed to you know, an idiot’s guide to how to get research done (U02)*

## Discussion

These findings confirm and extend the characterization of IKT initiation. All participants, including researchers, research users and connectors, acknowledged a distinct initiation phase of IKT partnerships. During this phase partners are identified and secured, and activities are undertaken to define the partnership, foster momentum, commitment and enthusiasm, and lay the administrative, governance and research ground work for ensuing collaboration. All of this requires intensive effort via multiple interactions over a protracted period of time, which ranged from 6 months to 2 years for 75.0% of participants in pre-existing partnerships, to 6 years for newly-formed partnerships. To facilitate IKT initiation, participating researchers and research users underscored the need for an IKT partner matching forum, IKT initiation toolkit or checklist, and funding for non-research activities required during IKT initiation to establish functional researcher-research user partnerships.

This study and its findings are distinct from prior research. Gagliardi et al. [2], Kothari et al. [22] and Rycroft-Malone et al. [11] identified the possible existence of an IKT initiation phase but did not characterize it. IKT syntheses described activities and determinants, but focused on IKT in general and not on specific enablers or barriers of distinct phases in the trajectory or evolution of an IKT partnership [8, 11, 33, 34]. Similarly, qualitative studies of researcher-research user partnerships focused on IKT in general or IKT phases other than initiation [17, 20]. For example, qualitative interviews with 17 health care researchers and research users from Sweden involved in 20 different projects specifically examined the later stages of collaboration [35]. Similar to prior IKT research, this study found that establishing and nurturing partnerships requires considerable time over possibly many years [2]. This study also found that partnerships are often based on pre-existing relationships [2]. A mixed methods study by Sibbald et al. including interviews with 49 researchers and research users also found that most partnerships were based on prior relationships [36]. Thus, while time-consuming, IKT initiation is an important activity that leads to functional partnerships, and if functional status is achieved, that is likely to lead to ongoing partnership for subsequent projects.

Participants offered three main recommendations for strategies, interventions or tools to support IKT initiation. The first suggestion was to create a forum or repository by which researchers or research users could identify partners with matching or desired interests or skills. In other research we interviewed research users from different types of organizations and they too underscored the need for a variety of forums, both in-person and technology-enabled, that could support interaction that may lead to partner identification [37, 38]. Further research is needed to find and describe examples of forums or repositories so that they could be widely replicated, or in the absence of existing examples, explore the desirable characteristics of forums or repositories. An alternative option would be to employ boundary spanners or linking agents that could connect researchers and research users [39]. The two connectors interviewed in our study described setting up in-person meetings or one-to-one conversations to broker partnerships. They also mentioned reaching out to a core network of other connectors to identify researchers or research users with particular interests. The impact of connector roles on research use and impact has been variable [40, 41], hence further research is needed on how best to choose, train and operationalize such entities.

The second suggestion was to develop an IKT initiation toolkit that offered instructions and templates for initiating and nurturing researcher-research user partnerships. Toolkits are an increasingly-used knowledge translation intervention [42], and in general appear to be an effective means of offering guidance [43]. While guides for researcher-research user collaboration are available their use and impact has not been evaluated [44]. Thus, future research should identify and evaluate those resources, and assess awareness, use and benefit of such resources. If not found to be useful, then a new toolkit updated with more recent research evidence on how to optimize IKT initiation should be developed.

The third suggestion was funding for non-research IKT initiation activities such as travel or meetings to establish partnerships. An international review of health care funding agency policies for support of knowledge translation found that no clear consensus or standards: approaches and mechanisms varied across region and funder type [45]. Strategically tailored funding opportunities (grants) were the most prevalent modality of support, and the most common strategy within those grants was the linking of researchers to research users. An example of this type of grant is CIHR’ Institute Community Support (ICS) planning grants which can be used for planning activities for community development and partnership building activities [46]. While researcher-research user partnerships were promoted by international funders, it is not clear if non-research IKT initiation activities were eligible for funding.

Strengths of this study included purposive sampling to recruit participants who varied according to a number of characteristics that may have influenced their views or experiences. We sampled to thematic saturation, in other words to the point where no further unique information emerged from successive interviews, which, in qualitative research, signals that recruitment is sufficient. We also complied with rigorous qualitative research methods [47] and reporting standards [29]. However, the interpretation and application of these findings may be limited by several factors. We recruited researchers and research users who were funded by a particular type of research grant predicated on partnership and from a national IKT network. Those individuals may be experienced in IKT and expressed views that might differ from individuals with less IKT experience. We interviewed only Canadian researchers and research users, thus the views expressed by our participants may not be transferrable to other geographic settings.

## Conclusions

Based on interviews with 22 Canadian researchers and research users involved in collaborative research, IKT initiation is a confirmed distinct early phase of partnerships requiring from 6 months to 2 years for pre-existing partnerships or up to 6 years for newly-formed partnerships. IKT initiation was characterized by identifying and securing partners through multiple interactions, and activities to define the partnership, foster momentum, commitment and enthusiasm, and lay the administrative, governance and research ground work for ensuing collaboration. The majority of participants represented long-standing partnerships. Therefore, initiation is an important early phase of IKT that establishes functional partnerships, and once established, ongoing partnership for subsequent projects is likely. Further research is needed to develop and evaluate approaches recommended by participants for stimulating IKT initiation including mechanisms for partner matching, an IKT initiation toolkit, and funding for non-research IKT initiation activities.

## Supplementary information

**Additional file 1.** Data extraction from interview transcripts.

## Data Availability

All data are available in the manuscript and the additional file.

## Abbreviations

ACC: Academic Collaborative Centres
CIHR: Canadian Institute for Health Research (CIHR)
CLAHRCs: Collaborations for Leadership in Applied Health Research and Care
COREQ: Criteria for reporting qualitative research
IKT: Integrated knowledge translation
PHSI: Partnerships for Health System Improvement (PHSI)
