# Supplementary material for: Initiation is recognized as a fundamental early phase of integrated knowledge translation (IKT): qualitative interviews with researchers and research users in IKT partnerships
Source: BMC Health Serv Res. 2019 Oct 30;19:772. doi: 10.1186/s12913-019-4573-4 (PMC6820935; doi:10.1186/s12913-019-4573-4)
Supplement: Supplementary file 1 — Additional file 1. Data extraction from interview transcripts. [file 12913_2019_4573_MOESM1_ESM.docx]

Additional File 1: Data extraction from interview transcripts

| Interview question | Theme | Researcher (R) Quotes | Research User (U) Quotes | Connector (C) Quotes |
| --- | --- | --- | --- | --- |
| Briefly describe objectives of research that you were involved in and your role | Objectives | This is a randomized control clinical trial aiming to determine the effectiveness of a new mobile device application. (R01)  Working with our nursing department on improving hand hygiene (R02)  Increase public engagement for cancer program decision-making (R03)  Inter-regional variation in patient flow performance (R04)  To evaluate the outcomes and implementation of managed alcohol programs in Canada (R05)  We were interested in trying to examine the integrated knowledge translation within each [5] projects (R06)  What are the barriers of the facilitators and the readiness of the population to uptake this [specific] policy? (R07)  Increase the capacity of frontline staff in acute care (R09)  Objective was to try to implement an intervention that would aim to improve pain management in the emergency department (R10) | We initiated on the basis of grants that were funded that had a pre-specified KT plan and then we worked really hard to connect them up to specific knowledge users; it was a homecare focused research project (U01)  Mobile app to track healthcare worker activities to assess healthcare worker's program (U02)  Creation of an implementation handbook introducing [healthcare professionals]’s into primary care settings and family medicine practice. (U03)  What impacts panels sizes in primary care? (U04)  Evaluating usability of a technology that can help support [specific type of patient] living in the community. Itt was a GPS locator technology (U05)  Working with researcher to develop a foundation grant award for researcher and knowledge user projects (U06)  We’ve tried to articulate that in a memorandum of understanding... putting in place some formal structures to enable that relationship to be built and developed (U07)  have interdisciplinary teams of clinicians who work with my health research methodology staff to identify and interpret the evidence and come up with recommendations (U08)  To support patient engagement and health system user engagement in our own programs internally. (U11) | So in work that were currently doing with the [province] is looking at an impact framework in terms of how research informs decision in terms of policy and practices, processes, etc. And taking kind of a similar approach that we are hypothesizing that in the very early stages if the researchers and the end-users identify the questions, needs and priorities using a code developed model and the chances of optimizing or achieving the outcomes and impacts will be higher (C01).  We have put funding programs together that have tried to support that kind of IKT approach and that relationship building (C02) |
|  | Length of initiation period | About six years (R01)  6 months (R02)  26 months (R03)  20 months (R04)  24 months (R05)  over 12 months (R06)  24 months (R07)  About 12 months (R09)  So depending on which users you’re trying to get too, obviously the time involved in trying to establish these relationships and these partnerships varies tremendously (R10) | 24 months (U02)  4 years (U03)  Six months to a year (U04) | Six months to a year (C01) |
|  | Identified potential stakeholders through workshops, pilot projects, conferences, meetings, and contacting pre-existing contacts, environmental scans | So when we commence new programs we had a stakeholder engagement process where all of the programs initiated out of the community based on an engagement process with the community in terms of needs and gaps and that process resulted in the development of all of our new programs…It involved workshops with the community in addition to a standardized needs assessment survey (R01)  Pursuing individual discussions with contacts that I already had through previous work (R01)  Okay, so I began by presenting the idea of the [Product Name] at meetings where there were both researchers and also knowledge users. And so I started through the venue I believe where it was a gathering already of knowledge users and researchers and you know weaseled my way onto the agenda so that I could put the idea out there and try and attract interest (R01)  Contacted pre-existing relationship (R01, R02, R03, R05, R08, R09, R10)  We did an environmental scan of some structural and population features for background and also some people would realize that this is actually moving along (R04)  So from the pilot data we had been able to identify the kind of the key players that would be partnering with us both from the patients side if you will and the health professional side. So here I mean the health professionals who work in the emergency department as knowledge users (R10) | Sometimes we need to pull in appropriate partners from the ministry or even could sometimes even be from another ministry that where there’s relevance…housing sectors there’s multiple ministries that might have a role. As well in housing we have a number of different organizations that we contract with who provide long term care and supportive living services. So we always need to look and see whose being impacted potentially. Who might have knowledge and expertise that is relevant and make sure that you know if they, if they’re you know quite closely impacted or you know would have critical input to give that they’re at the table (U05).  Or when we were sitting, we’ve been offering knowledge translation capacity building workshops for researchers and users. And there was an opportunity came up to actually study those workshops because there’s nothing actually in the literature particularly that talks about how successful it is to do capacity building workshops. And so we had a researcher who was very, who was offering these, also very interested and so we partnered that we’ll, you know we’ll be out there, we’ll help to pay and organize and bring the groups together and do our regular workshop stuff. But the researcher…do the training would also make it as part of a research project and study the whole thing (U06)  VP at the university and the VP in [Regional] Health coming together and organizing several sessions where the researchers and some of the decision-makers in [Regional] Health were led through workshops and that took a long time (U07)  Pre-existing relationship (U01, U04, U05, U06, U07, U08, U09)  We do a lot of knowledge transfer activities like national webinars you know blog postings like that and certainly that’s something that we have helped various researchers over the years at doing as well. (U09) | So it was all driven out of stakeholder engagement; all of our programs and that was a formal process, a systematic process and involved workshops with the community in addition to a standardized needs assessment survey. (C01)  And so not just to sign the letter of support but also to look at the applications in the early stage and say, yes this is something that’s of interest and yes, they have the right people involved. Or no, they should be talking to this person here and making the connections. So but what we’ve begun to do a little bit more recently is to more actively engage and be the connector between the researchers who are interested in something and the policy-maker or the clinicians or service providers that are the most appropriate to fit (C02) |
| How was partnership initiated? | Conducted research together as an extension of partnership | For many of them the partnerships have been pre-existing and…the research is in some ways an extension of the partnership (R02)  And so we ended up doing a pilot study together, designed it all together, like I really did very little, collected all the data together, analyzed the data together....there [were] focus groups involved and surveys, some data collection and you know they were the ones who sort of like recruited people and scheduled everything and coordinated it all. And then we did the focus groups together, we did the educational stuff together…And then we developed our intervention out of that work and sort of refined it and so on (R09) | I sit on an executive committee as the knowledge user for a foundation grant and that is regular meetings that get together with this executive group and looking at the business how to run the network and what that looks like and then how to inform moving those things forward (U06)  These people are involved from the out-set of when we start a project. So there are structures, there’s governance within my program within [Provincial Organization] at large and within sort of the [disease] field in our province which allows us to identify people and bring people on to the various, the individual guideline projects that we do (U08) | We identified; a) that it has to be a priority within the health system and there were some priority areas listed but they were fairly broad. And secondly, that there had to be both a policy-maker and health service provider actually engaged in the process (C02) |
|  | Contacted individuals with dual role as researcher and research users | So I’m thinking specifically back, I used a prior connection. I had people that I knew were not only researchers but they were also had some role in the [research user] in [Province]. So he sort of wore two hats; one hat as a researcher but one hat on the sort of advisory operations arm of [Provincial service]. So I used that relationship and that person who had this kind of dual role to take me from the medical side of things to the kind of operational knowledge user side of things and through that connection he introduced me to the non-physician operational people that would eventually support the project and become knowledge users on the project (R01)  On another project for instance, there’s a former colleague, she was a researcher, she’s now a decision- maker and when she left her research role we decided to go for lunch now and then and find out what some of the pressing issues were in her neck of the woods and for a while it was just this meeting for lunch but eventually that evolved into a project where she was talking about certain things that were a big issue and I thought okay, well this sounds like something we might study. Are there other people I should talk too and we built a team from the ground up that way. So she introduced me to a number of people I wouldn’t have known otherwise. We actually got funded, that project is just wrapping up (R04)  So was very fortunate to have senior mentors, who had been bridging this gap between research and healthcare system for some years ahead of me; who were able to help me to create a viable sort of feasible grant and to engage some of the senior leads to write as a support for this activity (R07) |  |  |
|  | Worked hard to convince and get partner buy-in on the research project | The Co-PI whose identified as the knowledge user PI on the grant partnered with me; was in the role of the VP of [Provincial service]. So that was my prime knowledge user but there were also other people who are not listed on the grant but just through the process of engaging with this, that person and through [Name]. There are others in the organization but then sort of you know I needed to get buy-in and approval from them and convince them that they would use knowledge from the project and the organization would benefit from that.. Well I didn’t have grey hair when I started it and now I do. (R01)  Like you’ve done all this work, you listened to the policy-makers, you’ve done this research together. And so you have this taddah moment, right? So here’s the results of this work or you know whether you’re building a toolkit or a framework or you know or whatever to help them. And so it’s like well, you need to keep on working with them to help them understand what that’s about and how it’s useful to them. And sometimes that’s the hardest piece because sometimes we don’t know either, right? And it’s still a work in progress (R03)  Then we did continued planning of trying to get me on the agenda [of the stakeholders meeting]…Not everyone initially thought this was something that they wanted to participate in. I had one region almost fall through. There were people that were interested and then a higher level manager said, no we don’t want to do this because we have no time. And I had to reach out to that person and explain this would be a very, very minimal burden on the participating regions that was another enabler to try to see incidentally (R04)  We’ve as I have said, I’ve had meetings where I was kind of the travelling salesman you know going to these various groups and trying to build these relationships… And so basically what happened is that you initially kind of think you’ve got the right people but then you meet up with them and they tell you well no, it’s not me but your project is great but it’s this other person. And so in the end you end up meeting a whole bunch of people and you having to kind of sell your project and try to convince them that this is something worth more than just endorsement but something that they would like to get involved with (R10) | Finding existing meetings to bring it forward and then you know at the end of the day the biggest argument is but my patients are special or I’m special. And at the end of the day we said everybody’s special, help us determine what we mean by special and we got buy-in (U04). |  |
|  | Initiated by research user | In the case, the grant we have right now that we were awarded in 2017, actually the decision-makers and policy-makers were the driving force of the grant (R03)  But yah, I’ve rarely have ever done a project that wasn’t responding some pressing knowledge need. It gets a little diluted when there are this many regions participating but it was certainly initiated from a decision-maker priority (R04)  And so when we did the initial pilot evaluation with one of our community partners you know we initiated that at their request (R05)  The decision-maker, my co-lead sort of administrative person, right? Came to me with the idea because they knew, you know I had some interest in the topic area; two of them were cancer related and that’s what I do, right? And the other one was sort of care of older adults and frailty and I do that as well. And so they came to me with an idea and they were all new, like they were all, I didn’t have longstanding relationships with these people at all actually (R09) | The project was initiated by [Provincial] Health Services. The researcher you know who was recognized ….in this field would have been approached to see if she would have an interest in being involved (U05)  Typically it starts with us… we have a partnership matrix that the executive team and the board have prioritized in terms of I guess assigning an executive lead in assigning like a project sponsor, setting up governance and we have formal participation agreements with those big partners in the system. (U10) | stakeholder engagement process where all of the programs initiated out of the community based on an engagement process with the community in terms of needs and gaps and that process resulted in the development of all of our new programs (C01) |
|  | Initiated by researcher | I was particularly interested to see well, if we’re trying to do this maybe it would be interesting to try to study it [IKT], not only encourage it but then find out how well it worked because they were different projects. And so I would say it was initiated by me just being curious as to whether not, how easy or how hard would it be to try to study it could we do that and then what kind of method would be appropriate to try to study it. So I thought about that and then had some conversations with [researchers] and others about just how we would go about doing it. And so I would say it was initiated by me and supported by the knowledge user at the time because I had a conversation with her about would this be something that would be interesting or of interest to her and her [portfolio] and there was support for that as well (R06) | I’m thinking about another big research initiative but again, I haven’t been so directly involved in myself. But where it’s a big sort of collaboration and that one was certainly initiated by the researchers but they have made an effort to engage you know folks from [Provincial Health Service] and the Ministry of Health here as well, as kind of decision-makers or knowledge users. (U05)  And the experience is very different from case to case as you can imagine. It really sort of depends on what the application is about, how much we’re brought into that. And a lot of it depends also on the researcher who approaches us as to how much they want us involved or at least how much we perceive that they want us to be involved, maybe that’s a better way of putting it. (U06)  We were approached by the lead PI and she had a massive PAN Canadian cohort of investigators that were interested in looking at remote tele-consult for specialist services (U09)  We often have been approached by health system researchers to, who want to generate knowledge that would apply to our programs and products but they also are looking for us to help them engage with patients in the courses of conducting their research and so we’ve done that in a couple of different ways as well (U11) |  |
|  | In person meetings | And I feel like that in-person meeting was when things really changed and I could feel that there was buy-in (R01)  And I also used the planning grant to go and meet in-person at each of the regions where we made a connection for the planning grant and also to see what I could do either in-person or by phone in regions where we haven’t made a connection yet (R04)  So at the beginning there was definitely where possible where I could meet with people. I chose to do that one-on-one where I could. The individual who was one of the key I would say knowledge users at that time before they left was in fairly close proximity to my office. So I could walk because of where my office was, so I could walk over and meet with her (R06)  So that face-to-face component I think always essential in building the trust, the human relationship part to move work forward across the multiple sectors and with people who don’t necessarily work alongside each other very frequently (R07)  Monthly meetings 1.5 hour each (R09) | Face to face meeting once a year [otherwise] teleconference or email (U06)  The president of [University] at the time and myself (CEO Health Region) had some exploratory conversations and there was some real interest in synergy there (U07)  I found more successful is at least to have an in-person kick-off … I feel like when you see somebody face-to-face you’re less likely to maybe ignore that email or have it fall to the bottom of the list of things … Like even if it’s a kick-off of a new project or something, just try to do it in-person, try to get, even if you have to fly to the hospital in [another City] to do it (U10) | So it really depends on the situation and on the previous knowledge of one another. I personally think that it’s really important to meet face-to-face (C02) |
| What activities or types of interaction took place during initiation? | Clarified roles, responsibilities, scope of project, research question by meetings, committees, agreements, | So the formal like legal contract of who’s doing what is just being drafted right now. But I think the expectations initially were laid out in a personal communications kind of informally and also in the letters of support and the grant application (R01)  So as much as possible the face-to-face meetings and being able to be clear about that their input is really valuable to this and what you see their role is (R03)    Scope document [took several meetings to clarify] what’s the scope, what’s the focus, what are the methods, and I summarized what I’ve heard from people, their interest, their concerns and I suggested based on what I’ve heard this is what I think we should do (R04)  So the first is the agreement about how will they be involved in recruitment, how will they be involved in data analysis, how would they, and then you know agreements about how old they’d be involved in or what do they want to do in terms of publications rather than a pre-set upfront agreement. We generally start with principles for how we’re gonna work together and timing you know frequency and timing of meetings. (R05)  Research charter or a project charter to help people feel that they’re clear about what their role is in association with the project and who else they might be able to call on when they’ve got issues they need to resolve (R07) | It started off as an ad hoc committees that became more structured committees which then became a formal research group (U03).  Advisory committee and I’ve sat on several of those where key stakeholders, not just policy-makers, decision-makers but also practicing stakeholders; are engaged in an advisory capacity throughout the research work. So the engagement then is very meaningful. By doing that, the knowledge exchange and knowledge transfer has actually been growing. It’s not an event (U04).  Well we try to clarify that at the very beginning just even when we’re asked to be part of a you know a letter of supports for an application. And it’s sort of what do you expecting from us to get a bit of an idea. So even if it’s just in terms of time commitment which is a huge deal because we’re all quite busy. So how much time are you expecting from us and then it’s part of it is just going through the process with them and getting a better understanding of where we can give more time. (U06)  And then we drafted a MOU and it went back and forth between the two, the two organizations; ultimately was discuss by our Board and was discussed by the [University 3] Board. And then the presidents and the board chairs of both organizations signed that off. And then we’ve used that to guide discussions between the two universities or between the two organizations since then. It you know I might say that it was really important at the beginning of building the relationship (U07)  So we use contractual levers, data levers, clinician engagement levers, policy levers. So I would say [Provincial Organization] as an organization is quite sophisticated in their use of partnerships and setting them up. And that was out of necessity because we’re usually doing fairly large costly and complex projects with our partners (U10) | We do facilitate meetings, face-to-face meetings where I’m included sometimes, particularly if people don’t know one another. And it needs, and it feels a little more formal at the beginning. But when there are people, two people who know of each other and know they’re working on something similar but what they need to be doing is connected I would send an email (C02) |
|  | Applied for a grants, wrote letters of support together | Yah and some of that relationship building was just happening through the creation of the research…just because that forced communication and so we were building, I think there was sense that by applying for the grant together that this was more than just some guy showing up with this kind of interesting new mobile app, just kind of wanting to show it off. (R01)  The act of actually writing the letters of support together was also a way that we kind of ensured that we were all I think on board with the questions that we were asking and the way that we were gonna get the data. So there were lots of back and forth. as far as those letters go and the protocol and the application and it was all over email but it involved a lot of comments and edits and they were probably five or six versions and it was the iterative process that kind of developed the project (R01)  Again the [GRANT] has been sort of, so I suppose we did have a bit of a relationship for a year because we did that pilot work. But before that, I had no…I didn’t know these people at all. And then we got the [GRANT] and again the [GRANT] was sort of completely, well completely followed our pilot work but was completely sort of developed together and the intervention has been delivered together and the data have been collected together and stuff like that. (R09) | Just you have this relationships that you just are, to me that’s the enabler to a great extent is we have good working relationships just in general. And when we get on a grant with them it just makes it easier to work (U06) |  |
|  | Aligned idea with research user organization’s goals: educating each other on how to align idea with organizational or research goals | They didn’t know about this innovation. So I had to, I thought my first role was that I had to introduce them to the innovation and teach them about the potential for this innovation to help them with their mission…. And then the part two of that was to seek their partnership on the actual research development itself (R01)  I think it’s really important that at what phase are you when you are initiating a project, at what phase is this particular research project in the context of other things that have already gone on. So I think the history of a collaboration is important because I think then you have to decide is this something that you need to re-engage people as in the case of new surgeons, you definitely did need to do that. And then how do you then keep this particular practice, community of practice important in the view of competing, I would say competing priorities because all the surgeons we talked too, they were all, some of them had academic appointments but a lot didn’t, we’re based in community hospitals (R06)  So with the work we’ve done, we’ve created steering committees where we invite the health system leads to review the research and the project periodically and to make sure that we’re remaining aligned with their priorities and to seek their advice on what else they need to know, where else we might be taking the findings (R07) | If a researcher came and said, hey let’s set some priorities up, I might say, you need to start with some of the key documents like in our case there’s [Provincial Health Priority Document]. You want to see what’s important to me, you go look at those documents and then come back and talk. So that’s part of, I find that part of my role is just educating researchers who want to make an impact on what, how do they engage in that conversation upon initiation because you might say, I’m really interested in some topic that isn’t even on the radar and you don’t line up to the you know the platform commitments or the budget commitments. (U01)  Engage them [clinicians] but inform them and educate them because there’s a huge distance between practice and policy (U04)  Priorities might be set by government or by leadership in the organization. And so we were looking to you know align with that or follow that (U05)  So when we’re approached we do look at it fairly critically to get enough information. What is the benefit to be on here? In KT it’s sometimes a little harder because we are all very much enthusiastic about promoting knowledge translation in general. Like we really want to see if people understanding KT, understanding a place in planning and science and being able to build the skills and use these provincially, nationally and internationally to you know increase the use of a relevant health research evidence. So everything that comes to us along those lines are things that we can passionately say yes too. But you have to step back and go, we can’t say yes to everything. So what is that actually, specifically we can improve what we’re doing as a foundation within this province? (U06)  It could be priorities emerging because [Provincial Organization] has to provide advice around funding decisions or organizational decisions. But in all respects or in almost all respects, they would also align with the [Provincial Action Plan] which is a plan that [Provincial Organization] creates every few years to direct its scope of activity and its priorities and what its function is gonna be(U08)  I would say, [Provincial Organization] actually takes a very, I guess robust or rigorous approach to partnership and it’s quite formal. But we found that that formality helps with very clear roles and responsibilities clear setting of scope and objectives for a partnership and they don’t necessarily need to be on-going (U10) | standardized needs assessment survey and workshops (C01)  SWAT analysis (C01) |
|  | Provided opportunities for communication such as events, and workshops | So we have some teleconferences, we do meetings, we send people email updates; for the [Local Group for Infection Control], we have education days twice a year where we bring other speakers in but we also use it as a mechanism for presenting results that people can think about. (R02)  So reached out to them and a clinician as well and they were very responsive. They attended our deliberation. We wanted them to know: a) what was going on in their backyard that we were gonna be there. We were gonna be doing this engagement event. We invited them to attend which they did on a weekend, it was awesome. And in fact, they’ve become, they are now on our new grant as stakeholders. So that was a really great relationship to cultivate and develop and they were enthusiastic about our work and they wanted to be part of sort of the next iteration of the project which is the one we’ve got right now (R03)  I think the opportunity was equal for all the regions because I did try to reach out to people to make sure everyone was consulted. And I also looped back to [Region] just before starting the data collection to make sure that whatever we had started off here haven’t been forgotten (R04) | Well now most when they were much more in-person in the olden days. The, that opportunity to participate as an external reviewer and the way that we did external reviews, that it was very transparent, that the committee would then respond to the reviews and make changes accordingly or to give feedback about why no changes would be made. I think that was a way of broadening the engagement to go beyond people who are just at the table (U08)  And she [PI] would host more than one meeting just to make sure that everybody felt a part of the discussion and dialogue. Email was used quite a bit too… There were some working groups established and that was of course…that we gave feedback on where we saw ourselves in what working groups we would want to contribute or participate as partners (U09)  teleconference and email just because we’re a provincial organization (U10) | So that would be a good example of a partnered initiative where we would facilitate meetings and workshops and kind of one-on-one discussions with the program managers and the researchers (C01) |
|  | Identified stakeholders | So in terms of who specifically the participants were going to be, the balance of strategic and operational roles, for example for interviewees or what roles exactly; those were things that were negotiated out with people, once we’d already decided we were doing this work but more of an adhoc basis (R04)  So we reached out, we found out who the [lead] was and we reached out to them to say, you know we know you’ve got a new program we’re putting in a big evaluation with other programs in the country…you don’t know us and we don’t know you but we just are gonna take and it was very short timelines but by the time we figured out about them and we just said, we’re gonna take a short stab and see if you might be interested and we know you know we’d have to work with you and get to know you and all that. So the interesting part of that story is the program responded to my email...literally within minutes, absolutely yes! (R05) | So quite often we have to think about engaging our colleagues who are more at the frontlines of the service delivery side of things. And as well, sometimes we need to pull in appropriate partners from the ministry or even could sometimes even be from another ministry that where there’s relevance as well even outside of the Ministry of Health in housing sectors there’s multiple ministries that might have a role. As well in housing we have a number of different organizations that we contract with who provide long term care and supportive living services and yah. So we always need to look and see whose being impacted potentially. Who might have you know knowledge and expertise that is relevant and make sure that you know if they, if they’re you know quite closely impacted or you know would have critical input to give that they’re at the table (U05)  think the other thing is willingness for people to understand the practice environment and develop those relationships. Some experienced researchers I think is needed. You need a range of research people with a range of research methodologies and sort of positions in their research. So qualitative epidemiology, quantitative, you needed that, that kind of range of perspective (U07) | And so we work, because we work so closely with our different stakeholders going you know from ministries and academics to health system or whatever, we will, we do have kind of a standard analysis and then part of that is in terms of moving forward to your point; is then using that based on kind of doing horizontal scans or looking to see who else is in the funding in the area; is other emerging areas, so and we get data from different sources. So you know looking at emerging areas in terms of for example, artificial intelligence. And so then that would help us explore new stakeholder groups you know depending on priorities. So and because we work cross-sectoral that stakeholder group analysis is broadening, (C01) |
| What factors enabled partnership initiation? | Leadership | Yah, I would say for us it would definitely, well leadership will be underlying always important. So it was mostly the operational leadership of [Provincial service] and a separate group was the [Canadian Charitable Organization] (R01) | So I think you need consistent leadership. Because you have a lot of turn in your leadership group that would make this hard. And there was stability at the university and there was also stability in [Regional] Health in terms of leaders who were working on this. So there wasn’t a lot of hand-off in between (U07)  Softer skills around not getting too fussed by that because also you have to do the flipside and really be praising and encouraging the groups and thanking them because you are thankful for what they do... So and that’s aspects of leadership, right? Being a leader in the good times and in the difficult times (U08) | I would say for us it would definitely, well leadership will, is all, will be underlying always important (C01)  so if key principles or key characteristics of the individuals, right? And you kind of see that just generally and it’s almost like it’s what you know comes out of leadership material or leadership training. It’s an openness to change and a willingness to hear others and making space for that open communication and having things adjusted in order to do that. And you don’t always find that with everyone. Like not everyone is good at that or find it easy to do. And I would say that not all researchers are trained in that kind of a way of thinking either (C02) |
|  | Shared goals between researchers and research users | [The protocol] document served as you know an understanding between us about what we were doing Because until we actually got those documents created it was all conversation and presentations and discussion but it was all out in the air. The letters and the protocols sort of crystalized what we had been talking about and confirmed that you know everybody had read it and looked at it and thought about it and I felt like that was the mechanism of how we actually got the projects confirmed about what we were doing together. (R01)  But I would say there is a process and the process is that you need to understand the knowledge user and what are they gonna do with the results. What do they want from you? What are, do they need to make their life easier? And try as much as you can when you’re thinking through about how ultimately you’re getting their input it’s obviously needs to be something that they’re really interested in or they’re not gonna consider it in their own portfolio. But I think you need to be able to think through about how best do you need to provide information to this person so that they have a better chance of being able to use the result or their team because often it’s a team (R06)  I think the research charter was more important for academic members than it was for health services members, that’s in contrast to a project I’ve recently done in that last couple of years which was the quality improvement project in a clinical space. That was a very different kind of project charter, much more created by the clinical teams for the clinical teams, much, not about, not a research charter but a priority charter around what was in scope, what we’re gonna change, what will we not gonna try and change during the project. And that one was created by the clinical teams themselves, so very different for different purposes (R07) | We set the parameters, we set the priorities that was based on alignment to government priorities and that’s how we set the funding call-up. So explicitly we asked researchers to align and then we had a whole relevance review with policy colleagues and they, it was a two-stage review we call this. So the policy colleagues would rank it in terms of its policy relevance and then a scientific panel will rank it based on scientific merit (U01)  Identifying the common concern and identifying what everyone’s goal truly is (U03) | Strategic alignment, so is really wanting to partner with partners who are trying to achieve similar outcomes (C01)  So we have found a core group of people that we will always go to and if they aren’t the right ones they will filter that out and help us. Those are the ones that we know we will be able to actually establish some connection with because they have identified an interest in research and you know that they think that it’s important. And the same thing goes on the researcher side that there has to be a willingness and an openness to actually work with the policy-makers and not all researchers are that open all the time (C02) |
|  | Form partnership early on; Collaboration from the start and throughout; | I think you really, really have to do a good job at pre-initiation as well as when you actually initiate the research (R05)  We established collaboration at the beginning inviting people to understand what we were after, eliciting their support and then thinking through about right from the beginning where would we disseminate our results too and who would do that? And so when I think about some of those things we didn’t, we had agreement on the study proposal. So everybody talked about that. We had involvement of our study team throughout and at the end. (R06)  Collaboratively from beginning (R09) | So you know a really forward thinking research team will try to approach bureaucrats and try to do that very early co-design but it is enormously challenging mainly because of time availability of the policy-makers (U01)  it would be nice if some people first start thinking about these applications and asking us if, what we tend to get is like a you know a one, maybe two page kind of summary of what the application is gonna look like. But you know it would have been helpful if we’d been brought in sometimes even earlier. So for example, I know…there was one we recently approached on that they’d had a large webinar for a particular project for the funding program. And I mean the researcher, maybe you can’t always think ahead who you want to have as part of your group. But if we could have been part of that, it would have really helped to set us up and understand much clearer what they were working for and what they were doing. Where there are opportunities to bring in us in earlier, I think that would be really helpful (U06)  So I refer to IAP II, the International Association of Public Participation. If you look at their framing it forces you to think about what are you meaning by partnerships? So if it’s strictly to get endorsement that’s one thing. Is that equalled decision-making then you’re wanting a different level of relationship with me. If you’re wanting feedback that means you’re doing the work but I will comment on it. So I think the expectations at the on-set, the intent needs to be very clear (U04)  And then we drafted a MOU and it went back and forth between the two, the two organizations; ultimately was discuss by our Board and was discussed by the [University 3] Board. And then the presidents and the board chairs of both organizations signed that off. And then we’ve used that to guide discussions between the two universities or between the two organizations since then. It you know I might say that it was really important at the beginning of building the relationship. It’s probably less important now and it’s probably referenced less now. Although we do still pull that out and we refresh it but it’s, you know as the relationships have at the organization level have been established, its, so it was really important at the beginning I guess is what I’m trying to say (U07)  Make some deliberate decision-making around our priorities where we can invest our time and effort and that’s sometimes hard to do and maybe that’s something we could be a little bit more transparent about; where we are willing to support researchers and have those conversations earlier (U09). | As an organization we would see ourselves not only as a funder but as a facilitator or a convenor or a catalyst for bringing together at the very beginning as part of needs and planning to kind of address shared needs and moving forward. So yes, both our organization and our department. So the term we are or our practice is moving very much to a partnered... or partnered approach in addition to co-developing right along with our partners through need planning development and implementation (C01)  But to have, to try and establish those kinds of relationships early on does make for successful approach at least. I think it doesn’t where I’ve seen things work that’s where I’ve, where I’ve seen it work. Those relationships are there, they’re not just created because of a specific you know fund or whatever (C02) |
|  | Trust: Demonstrate commitment to the project, personalize emails, be responsive, face to face meetings, maintain credibility | I think there was sense that by applying for the grant together that this was more than just some guy showing up with this kind of interesting new [product], just kind of wanting to show it off (R01)  So what it meant is a personal email for me…phrased in a very specific way. I have to think about what is the title of the email that will get their attention. I make the email relatively short but I make it extremely personal. If I know anything about them, I say something about that. If I don’t know anything like when I reached out to the person I told you that you know we approached late and then she was very responsive I basically started my email with saying you don’t know me, we’ve never met. I just want to tell you who I am, what you’re doing and I can tell you one hundred percent when those emails come to them probably one of the first things they do is they Google you…to see who’s that researcher. They look at what you’ve done, they’re looking for, they’re looking people who they think have some of the same values. They might even ask their colleagues about you, right? So your reputation really matters. And if you don’t have a reputation that’s fine, I mean you don’t have to but the way you write that email…Your priority has to be responding to their email, if they choose to respond to you and you have to make sure you make yourself like in my original email I’ll say something like, if you prefer to talk on the phone I’d be happy to do that (R05)  I showed respect that I reached out to them, that it was, I made an effort to come and meet with them to show my interest and engaging their support because without their engagement this really would not go anywhere. And so I think that that part is really important in reaching out as much as you can (R06)  So that face-to-face component I think always essential in building the trust, the human relationship part to move work forward across the multiple sectors and with people who don’t necessarily work alongside each other very frequently (R07) | So the president and I met several times. We also did went back and forth via electronic means on the actual document itself. When we did the signing of the documents or first of all, we took it to our respective boards; that was an individual process and then when we worked together on signing it and getting it signed-off. And then I think if I’m remembering correctly, we actually did have a kind of an event around the signing of the final MOU (U07)  And the other piece that is a really important factors is credibility. So I’ve always said to folks…the world is too small. I mean it’s not [City] it’s the whole world is way too small to burn bridges. So and I mean I can always tell you, I mean you know so I live in [City] but I do work nationally and I do work internationally. And if you burned a bridge somewhere or haven’t considered voices or haven’t followed through credibility is lost. And the other thing is, is sticking to the plan. So to engage people and then they don’t hear anything or there is no feedback or there is no cycle of feedback they will quickly become disengaged. You have to look at what the initial engagement is that you have to deliver (U04)  But a lot of this is coming down to civility. Like how civil are we being with our colleagues? How do we set out a very clear scope of what we’re actually trying to accomplish; people are so busy. People don’t even have time to read documents before meetings or anything or be prepared anymore (U10)  And so [Researcher] would provide regular communication and updates without necessarily asking anything of us. And so I just thought the sequencing of the communication she offered, the different levels of involvement and the different level of that sort of involve, yah making us feel like we were a part of it; I think was really good, well done (U11) | Sometimes we need to be patient in our timing, timing of applications in order to let some of this relationship building actually happen. So you can’t just you know in a three or two month period, right? That you’re writing the application form that you’re you know trying to find people that you know to engage in this conversation. You can’t just establish a trust relationship at that point… make sure that you can give enough time to have a trusting relationship because that becomes critical in the IKT process I think. And a factor, I mean I don’t know this happens. You know I don’t think this has been necessarily born out but that, if that trust isn’t there then you don’t get the kind of impact that you might at the end of it. (C02) |
|  | Pre-defined network in advanced of project deadline: Individual or organizational capacity to develop and maintain a network | I guess a piece of that [beginning a partnership] is about knowing whose gonna say yes, and not approaching the people who are gonna say no (R02)  So we always have the steering committee [we] keep them up-to-date. We speak with our stakeholders on the steering committee probably three times a year. (R03)  I was already embedded in the decision-maker context; in that people were hearing from their own colleagues in other parts of the country(R04)  Researchers really have to know the networks and they have to be able to tap into networks at different points in order to get a full picture of who is involved in this work and who needs to be involved in this work (R05)  And so I was aware through my work [previous project] that there was a group of people who were part of a drug user organization who were specifically focused on the harms of elicit alcohol and that they were advocating for programs for their peers. So I reached out through my network to individuals that I knew that were working with that group and they become like an amazing partner. (R05)  So I would say in both projects were really important that the PI was known or you know of the person you wanted to collaborate with through a previous experience. I think it’s really hard once you identify people if you’ve not come in contact with them before (R06) | Anybody who is in this space of health services and policy research really needs to have a network and they need to either initiate that themselves or they need somebody in their department whose role it is to help facilitate the creation of the network. And so again, it goes back to this idea of organizational capacity on the delivery planning policy side and on the university side because I’ve seen really effective professors who just are great at developing networks and those that are not and you know it feels a little inequitable that because of you know personality or you know introversion versus extroversion that you’re, you should not have that network that your colleagues does because they are more inclined to be you know meeting new people and expanding their network (U01)  It’s getting the group to gum up to speed and feel like a group that over time we use the project as a means to sort of create that sense of community and commitment and I guess that sense of you’re doing it together (U08) | Looking at those areas [of interest] and priorities and identifying who we should be partnering with that we may not have considered (C01)  From a researcher perspective to find, find some key advocates within the health system for your research and then they can always be there to help connect, right? And that’s not something that happens because of an application. That just happens because you’re, you know interested in doing something and somebody shares that interest and then you can you know sort of go on working on something together. So I think that that’s an important, that’s important piece and this trust and in being able to move things along in this kind of approach (C02) |
|  | Shared interest, synergy and passion for the topic | They are you know we’re empathic clinicians and we see when the care that they’re providing just doesn’t seem to be meeting the needs of the people that are living with the illness. And so it was not difficult when it’s a topic that people are internally motivated to you know the what’s in it for me component; is maybe it isn’t an identified selfish…as in what’s in it for me. It’s always identified as what’s in it for the patient (R07)  Yah, so I think what helped is that it was their idea. Like this is something they were very passionate about and they just needed a researcher who they could work with and who sort of you know who could bring that research lens and bring some of the rigour and they thought their work would be more credible and so on. But this was something that they’re really, really interested in and passionate about, right?... having them really like be passionate about the topic and coming to me with the question and I had to help them refine it as a research question (R09) | We have kind of a synergy around shared interests definitely yah when there’s sort of a number of stakeholders who are seeing a potential benefit or a you know a key gap in knowledge that there’s agreement this needs to be addressed with sometimes you know that aligns quite nicely with a researcher existing interests and quite often it seems to come together that way (U04)  Certainly you know sometimes we have kind of a synergy around shared interests definitely yah when there’s sort of a number of stakeholders who are seeing a potential benefit or a you know a key gap in knowledge that there’s agreement this needs to be addressed with sometimes you know that aligns quite nicely with a researchers existing interests and quite often it seems to come together that way (U05)  And so doing things that build up that quality relationship that and that kind of identify the areas of mutual gain I think are enablers (U11) |  |
|  | Funding opportunity legitimized and enabled researcher research user collaboration | So that CIHR funded body was really helpful in being able to find contacts and it also lent kind of legitimacy to my position within that network and their positioning within the network. So it’s sort of a, there was an automatic shared interest even before we met on another. So I would say that sort of facilitated the initial meeting of the right knowledge user because they were already at the table to discuss things within the general category of improving survival from cardiac arrest (R01)  I would say the grant opportunity itself. So you know the opportunity to have the funding as a goal kind of brings people together for the purpose of the research. Whereas if the funding wasn’t available this relationship would have never happened to be honest, so yah the funding opportunity itself, like creates these knowledge user and scientist relationships that would not otherwise have happened as easily. (R01) | Funding opportunity that’s well aligned (U05)  Just you have this relationships that you just are, to me that’s the enabler to a great extent is we have good working relationships just in general. And when we get on a grant with them it just makes it easier to work (U06)  Think the other enabler and this was pretty critical is the [Private Foundation Grant] had some, had an investigative team grant process that they were unfolding as well as capacity building grant for health authority that they were unfolding. And we were successful in getting an investigative team grant that was a collaboration between the university and [Regional] Health. As well as a capacity building grant that enabled us to put some leadership in place at the health authority which we then, it was 3-year funding and at the end of the 3-years we sustained that funding into the future. But that injection of funding was really helpful (U07)  we’re a very well-funded guidelines program, sort of relative to most guideline programs world-wide. So the fact that we have resources and an exceptionally well-trained staff to do sort of the core work, that makes it easier (U08) | So I would say that is kind of happens with the funding. So an example is that with one of our funding programs, the partnership in research and innovation for the health system, [name of program], it’s a partnered funding program between [province] and [health system organization] and we have at the beginning really co-designed the program to meet the health system need and then have facilitated meetings and workshops with researchers and where the program manager would meet with the research community and the health system every quarter. And in terms of that partnership through that whole funding period. So that would be a good example of a partnered initiative where we would facilitate meetings and workshops and kind of one-on-one discussions with the program managers and the researchers (C01)  Well I, I mean obviously funding does help. So the opportunity absolutely, like the opportunity of funding and say just as an example this rewarding success. It provided an impetus for researchers who may be interested in doing something you know to really go out and talk to the policy-makers about their ideas and about what they would like to see done and vice versa (C02) |
|  | Funding for travel or other opportunities | The funds to make the face-to-face visit, I had done through my university through a research initiation grant. Which specifically you know could fund things like this which was you know a flight and trip for a few days from [City] to [City] to meet people in-person and so that was an enabler for sure (R01)  And then it’s a about finding places where we have the current group of people and the funding agencies and the practicalities allow you to answer a related question. And in my life, it’s a, say relatively complicated mix of what people will pay for and that’s from funding agencies to you know of all sorts to sometimes to companies that you know have an interest, right? There’s a, there’s some things that I would like to do that nobody is gonna fund, okay and there’s no point…about them because it doesn’t matter what kind of grant I write, it’s not gonna happen and periodically that changes (R02)  Money to meet in person, resources (R05) | Availability of resources to pursue the research (U01) | So it really depends on the situation and on the previous knowledge of one another. I personally think that it’s really important to meet face-to-face (C02) |
|  | Connectors, Boundary Spanners, Mentors | So by knowing both of those things they can kind of mentor you or guide you to the right people. And I really think it’s an advantage over a cold call of a scientist to a knowledge user that often doesn’t pick up and take off. But if you have a matchmaker then there’s some trust and sort of prior history, prior knowledge of you through this person that makes the relationship much more to be successful I think. That’s right. And often these people I find who are connectors like have their hands in a lot of pots are often mentors and in my case that was certainly true. So they’re often senior members I find. (R01)  Identifying people who have a leg in both camps. So finding those people who have like kind of live in both worlds, they’re great connectors. I think they act as translators and connecters. So they can hear, they can hear about an innovation or an idea or a project and because of their roles as both you know scientist and also knowledge users in an operational agency, they have a good view of the landscape of an organization and the politics of an organization and they also understand the clinical aspect of what you’re trying to do. (R01)  We had to, there was a lot sort of checking in that the research user wanted with the project; how are things going? Do you need anything from us? We want your principle investigators to do a presentation to [National Association], can you set that up? So that kind of stuff would all go through me and I think that’s a good point for them to have where they don’t, might not want to bother the PI or not sort of those kinds of organizational things, those kinds of checking in things. The big ticket questions they’ll go obviously directly to him but I can also mediate that at little bit. I can solve a lot of stuff for [Supervisory] or for the research user. So having that kind of point person who’s right on, that that’s a go to person for the research user, if they need that. (R03)  She [key stakeholder] helped us introduce me to the [CEO Forum of Regional Organization] and actually before she introduced me to the CEO Forum, she helped us make some contacts (R04)  My experience with a lot of kind of those knowledge brokers is that they some, they don’t have the networks but they’re excellent once you connect them to those networks….Anyways, they are really good, they were really good at tapping into some people that weren’t in my networks but were in their networks (R05)  You might not know who the people are so you need someone to help, help you understand the organization and it might take you a few tries before you actually get to the right person…You may need to use an intermediary, someone else to help broker that relationship initially… So the researcher is well known and they already have a reputation as being a good researcher. I think from my perspective on the first project I mentioned I think it…easier and our data from that study suggested that people were willing to collaborate because they really respected the PI, they’d done previous good work before and so they were willing to engage with them again (R06)  So I went to at the time my division head, and he had done work before in pain and large grant, he’s had success with large grants around pain, international work and very much connected to both the research community within [Province] and to the [clinical network]. So this mentor was really essential (R07) | There’s the idea of the boundary spanner role, you know that somebody who spent time in multiple domains (U01)  The chair of the nursing program was a key person in this, in this process; very experienced researcher and have a lot of creditability in the practice environment. And so she was, there were a few champions amongst the researchers that were really important. She was one. Another one was the person who was in the associate dean research position with the [University] Medical program. He was a very critical piece of bringing this together and really been nursing person and the medical, the research associate dean; the two of them were the ones that really spearheaded this. And then got you know people from medical geography, a social work and so on. The indigenous health, some of the other areas of the university to come to the table, so it was very definitely championed by a few key people (U07)  I sit on guideline panels when something is going a bit squirrely or something is [a bit] off. But if you have someone sort of a little bit removed, so when there’s a challenge or a problem or a difference of opinion, you have this sort of unbiased person, I’m not a physician, clinician, so like I’m disease diagnostic, I’m program diagnostic, all of that, that can help come up with a consensus of a remedial solution (U08) | Well I think it’s kind of having a foot in both worlds, right? We obviously very deliberately connect with the researchers and the research community to understand what their needs are and to just understand where the strengths are and what’s going on. So we do that as part of our role. But another part of our role as I mentioned is to ensure that we’re getting the greatest impact out of the research that’s funded so that there is actually translation of the knowledge whether that’s in health services or whether that’s a products being developed or you know policy change because of an engineering discovery or whatever it is (C02) |
|  | Time commitment from busy knowledge users: Try to minimize their time commitment | So a big piece of it is trying to organize the project so that you have minimal impact on clinical function, or organize it such that there is an actual benefit, the clinical function so that people are you know and then people do the research because there is a direct benefit or at least not much of an issue for them. You can’t go to primary clinical people and say, you know I need you to spend one day a week doing research to work on this research project for the next five years, right. It’s not gonna happen (R02)  It’s really important to be committed to taking as much off the plate of the researchers as possible, okay, or of the sort of clinician or knowledge users as possible. So you know to the extent of their administrative things that need to be done, data-sharing agreements, and contracts you know all of those things, paperwork (R02)  During the preparation of the grant I did a huge amount of admin work for everybody. . I don’t know how much that was appreciated but certainly taking that load off or at least removed a barrier to people (R04)  And pre-initiation is tricky because you’re basically asking people for information, you’re asking people for letter of support, you’re asking for their input into proposals and you don’t know if you’re gonna get that money…So the time and energy that we’re asking of them in the pre-initiation phase is actually quite huge. Plus, they don’t, if they don’t know you they’re not gonna trust you (R05) | So I think it’s being mindful of your stakeholders; what their day jobs are because really you’re asking people to be engaged off the side of their desks. So I think we have to be very mindful particularly in the current environment (U04)  You have to think broadly about how you can achieve the goal, the overall goal that you have while minimizing the impact on their time and making sure that their time is used wisely (U08)  Like I worked in an academic space as well as in the operation side and in academia there’s still space for like deeply understanding something and then having a conversation about it. That just doesn’t exist in the business world anymore (U10)  Being asked to do things at the last minute which I would say is a typical way that things occur because at least the way things work is somebody knows somebody, passes on a request and then you, there’s a sort of chain of having it passed on and it lands in your lap and you want you know comments or a response within a few days. And I have many days that are just back to back meetings so it’s very difficult to respond in a short timeline (U11) |  |
|  | Making research users feel valued and respected, not just a token partner, sense of ownership | A big piece of what makes clinicians I think willing to be part of research enterprises is having their input valued and having their contribution valued. And so, there’s two part thing, right? There’s a piece about getting their input which is valuable and then there’s the piece about making sure that you are always recognizing their input and valuing it and so, and that, so that’s about being willing to provide information to them if they need it… it’s about personal relationships and about people feeling valued and about people knowing that the research group is there to help them too (R02)  Sometimes, a researcher might have a very pre-defined notion of what they want to study and then goes searching for decision-makers to involve perhaps in a slightly tokenistic manner and there’s increasing pressure from grant agencies to involve decision-makers but sometimes what that can lead to is involvement that might take place around the question that even if it’s of some interest to the decision-makers won’t necessarily will acknowledge that’s a priority for the system (R04)  You have to reach out to your partners, when I say reach out I don’t mean the RA reach out, the RA who’s helped you write the proposal, no. It has to be someone who’s in a lead researcher role, someone who has the skills and the knowledge to work with community partners. (R05)  Once you do get to meet with them, I think you need to be able to present the case that their input is really important to what you’re doing. So I think you have to make the case and if you don’t make the case I think that that’s potentially a barrier (R06)  Never being quite sure to what extent senior leads within a health system are truly engaged and supportive of work versus yes you go ahead that sounds fine, I’ll sign-off on it. But you know don’t really want to be necessarily bothered with some of the findings that might require change or investment or you know so you’re never quite sure when people sign-up to say, yes, yes we’ll act on the findings or we’re supportive of this piece of work. Whether it’s gonna be something that’s at the end of the day they can act on or will want to act on (R07)  We all bring expertise to the table and no one is better than the others or no one will value one person expertise other, well I value their expertise probably than I do my own. But you know they come like so I bring research methods expertise or study design expertise but I mean they bring a whole lot of expertise and the inside knowledge and who’s who and they can get people involved and so on (R09) | I am feeling that partnerships are becoming token. So when we say partnerships they’d better be meaningful. If they’re not meaningful or if it’s a partnership to put on paper so you can get grant money, it doesn’t take a few of us too long to figure out that my engagement is of absolutely not important to you but was important to get the grant. So we need to really define what we mean by partnerships. Do you really want my involvement? Are you using my involvement to get money? And what is the nature of the partnership? Is it one of consultation? Is it an equal decision-making? (U04)  In other [instances] there’s hardly any interaction at all and you do feel that so you’re being asked because the researcher needs to show they got knowledge users on board and you kind of wonder you know. You know, hello, I’m here. Are you gonna use me or not? (U06)  I also think and I can think of several researchers that we’ve partnered extensively with who have probably sacrificed some publications because they haven’t invested their time in not necessarily disseminating information through a publication but they’ve invested their time in ensuring that the knowledge exchange happened right into the practice environment (U07)  Making sure that their time is used wisely and that they feel valued for their time…be nimble enough to allow that process to unfold in a way that the partners that we have, have as much control as they can around shaping, designing and thinking through what the evidence means… people feel ownership of that project and allow that project to unfold in a way where people feel committed, people feel good about the end result and because if they feel good for the end result, that’s gonna be also very useful for implementation (U08)  But certainly I think also that you know having a name of an organization or a logo and the creditability that we provide on an application. I think sometimes desirable for some investigators which we want to make sure that we’re meaningfully you know able to contribute and that we also are in agreement with you know the actual proposed objectives for the research as well so that we’re committing our time appropriately. (U09).  So it’s about truly ensuring that you’re understanding that person, you’re understanding their objectives out of this. What’s in it for them? But I think there’s a lack of, the lack of civility that happens with some of our more passive communication methods like teleconference and email. People are quick to be a bit more or bit less diplomatic I would say (U10)  And so, and would provide regular communication and updates without necessarily asking anything of us. And so I just thought the sequencing of the communication…making us feel like we were a part of it; I think was really good, well done. (U11) | Where I’ve seen it work is where there is openness on both sides, right? There’s…there is a real willingness from the researcher to engage with and openly dialogue with you know the end-users or the patients or the service providers or the policy-makers that that openness from the researcher absolutely has to be there. And if that’s there then it does allow for a lot more open dialogue and the you know the people who are engaged in the process can see value in what they’re doing and are much more willing to I think engage in an on-going fashion when that happens. I think if it’s just and most people can tell when you know the consultation process is not real and people already have the answers you know already accept (C02) |
|  | Have a good track record by being respectful of research users’ time, being honest about goals | You know making sure that we’re very consciousness about people’s time you know for these conference calls for any asks that we have of them; if we want them to, I don’t know, review something or take a look at something or ask their opinion on something. We’re very respectful of that, of their time and their interests. So that I think is key. So just sort of that, that interaction with them to be, yah, mindful of their time and yah, be respectful and not, if we haven’t asked make sure it’s a good one, make sure it’s sort of targeted that they have any information they need. It’s you know packaged into you know some bullet points that you know they could take a quick look at, ten minutes before the call so they’re brought up to speed, that we’re not wasting their time by giving them lots of background and you know just like you know executive summary (R03)  But you know I’ve got this idea for a research project and I’m gonna tell you about it right now and I always say, well you know if you think this is not relevant or whatever I’m like just tell me right away because I won’t do it. And lots of times their mouth drops open and I’m like no, seriously. If this is not relevant to you I’m not gonna waste my time trying to put something together, right? So sort of take that approach and I’ve said that to lots of people over the years and so now people know me and they know I sort of do that. But like I’ve had like I remember one guy, he was like head of diagnostic imaging because I had this idea and he was like but you’re a researcher you can do whatever you want to do. Like you know what I mean? I’m like oh no, I don’t…but I mean if this is not relevant to you guys here on the ground then I don’t want to do (R09) | The other biggest barrier and I’ve got one I’m dealing with right now is our expectations. If the research is expectations exceed the time capacity of the individual, it’s a huge barrier and that’s the biggest one. So scheduling meetings without enough lead time, keeping in mind you know especially if you’re a practicing clinician you got a busy practice or appointments but also thinking you’re gonna schedule a meeting in two week’s notice, is just not appropriate (U04)  I think it would be very helpful though if there was a bit more upfront discussion on what expectations are. I kind of feel like sometimes we have to kind of make it up. It’s like so, here’s where we think we could help but we actually don’t necessarily know enough about your project. Or could you tell us where you see using us in some ways. It’s not always clear what that looks like. Sometimes it is, often it’s not (U06)  You have to think broadly about how you can achieve the goal, the overall goal that you have while minimizing the impact on their time and making sure that their time is used wisely and that they feel valued for their time (U08).  So she [PI] provided a few different options around where we might want to contribute and instead leaving it to us, not to do off the side of our desk, to say this is what we can do. She would actually provide some templates or even you know it was pretty much like you said, like almost a bit of a description about here’s what I heard you say you could do, does this resonate and she would confirm that with us. So even you know helping support the development of the template letter of support on the application (U09)  And so I think that from the very beginning being explicitly clear on mutually beneficial objectives and then not allowing scope creep unless it’s all of the mutually understood and respected and formally added and keeping civil with you know difficult conversations in partnerships (U10) |  |
|  | Speaking the same language, shared culture | Also I’ve been around the decision-makers for so long that I sort of know the management language associated with this domain and even though I’m not a clinician I know some of the clinical language. So we didn’t I think have language problems going back and forth (R04)  Being able to talk in each other’s language and I’m a bit, I probably am not as good as I used to be at being able to talk in non-research speak (R05)  An essential change agent, who also use the language of what motivates the health service people knew the if you like, the buzz phrases of the day in terms of you know learning organizations and how to mobilize health service leads around a project by aligning it to their existing burning platforms or existing priorities (R07)  And so in terms of you know approaching if you will, as I said before kind of fellow healthcare professionals approaching middle management and even approaching higher up healthcare administrators; we kind of speak, everybody speaks the same language in terms of we’re trying to improve either healthcare delivery or we’re trying to improve you know management of some kind of issue. And so everybody understands that. So that you know the higher level, they’re more interested in cost containment maybe but we can use that language as well (R10) | First of all, it’s a practice driven environment even though research is an interest for lots of the practitioners and decision-makers in the health authority. The real drivers are about care and service delivery and it’s much more of a traditional organizational structure. Whereas at the university, it’s more of a collection of faculty that you have in a university environment. And the drivers are very much they drive people to individual work. Where what we need in the health authority is more of a collective endeavour. So and then the other thing at the university is often the drivers push you towards curiosity driven research and there aren’t as many incentives to encourage a robust knowledge exchange strategies (U07)  We talked a lot about what our, harmonizing of our ethics processes. So the people that were involved in those research review processes were involved (U07) |  |
|  | Research project as a way of research users to stay up to date or researcher to obtain tenure | They [Research Users] were very interested in being up to date and keeping in touch. And so particularly some in the community hospital saw this, a participation in a research project as a way of keeping up to date. So I think there was some motivation from their side that this would be one way to from them learn more about current practice and make sure that their own practice was up to date [R06] | [Outputs] can be published or they are published. So they’re always published on the [Provincial Organization] website of course but they’re also published in peer-reviewed journals...So I write many letters of promotion for clinicians moving to the next stage of their career and to ensure that that work and the website publications are counted as peer-reviewed publications. So some of those small things to sort of help the deans of medicine or the deans of health science and to understand what the work is and why we view this as peer-reviewed as much as when it goes to a journal (U08) |  |
|  | Geographic proximity | [Province] is really small, so we, you can always find a connection almost to sort of build the relationship even if it’s like you know your kids go to the same school or something like that. I’ve been really lucky out here because there’s a lot of informal, like everybody, I say everybody knows everybody and that sounds funny but it almost feels true. And physically, like geographically, physically we can meet more often, right? Like quite literally you know we’re all within you know I say we, like any decision-makers I work with or any clinicians for the most part we’re all within ten minutes of walking to each other (R09) | [City] is pretty small and depending on the question you tend to know who [to contact]. Often you know you got your key informants and it’s a bit like a web…limit the number on an advisory because you know people are not gonna come even if it’s every three months. So it’s usually between 15 and 20 people. Although often be invited as you know priority informants and then is there anybody we’re missing. So it’s not like we’re sitting in [large city] (U04) | In [City] is a small place and they know it’s easy to say that if you’re in hip and knees, this is the person that you should be talking too: So that’s kind of how we had done it. But you know where, yah I mean that’s it and I think it’s partly the system, right? That we because we’re so small we know everyone and we have the ability to identify who the appropriate people are and then make those connections. I think that an environmental scan and some sort of static document or even something on the web doesn’t always lend itself well to making these kinds of connections. I think if we were in a bigger place we would still need to play the role that we would play. We might just have to go down a couple of different levels and talk to a couple of different people to get to the right individuals (C02) |
| What factors were barriers to partnership initiation? | High turnover of stakeholders | When you know an organization like these, they are kind of reporting to their political master, their masters at the provincial level and you know those cycles are you know four years or shorter for our representatives in the government. And so I find sometimes they operate on like government cycles. And so sometimes when a government changes, like it did in [Province], it put a real sort of change in appetite for the project into the knowledge users. So this might be specific to this project but I think it was a barrier for this and it might be for other (R01)  The other issue of course is there’s turnover, like as this project is way, way longer than I’m used too. There’s some regions where they’ve already switched over three times, who that individual is and they may not be as involved as the person who started there. (R04)  But the knowledge user in fact changed and that certainly is an issue. So the person I spent some time talking with and developing a relationship with has since gone so (R06)  Where I have had problems before on-going and I can’t even think of, I have no projects right now actually where anybody from like the government is on like Ministry of Health but I have in the past and I found that always very challenging. And the barriers I think involves many things. One is I think like high turnover often in ministry. So people take different positions, right? They move a lot. And I think within policy-making, like policy-makers and at the ministry they’re you know priorities change quickly sometimes, their timelines are quite different than mine and there’s a lot of turnover and I think all those things have, I found it really difficult. Like it’s never been a bad situation or anything like that, it’s just really sort of it’s a barrier to sort of doing this, doing this work (R09) |  | So to keep sustained relationship with some of the organizations it can become challenging when there’s a high turnover of people (C01) |
|  | Over-reliance on one person representing a group of research users | I felt that the partnership kind of, the partnership between me and that knowledge user organization suffered a little bit because it all of a sudden became dependent on that one link, that one person (R01)  There was a lot of peer influence at all levels including the CEO level to encourage other regions to participate and to communicate the benefits of the research (R04) | But I would say that a lot of those partnerships and gathering of those folks for the different committees, I’m not convinced we have been completely representative of all of the clinic, if I focus on the clinicians specifically; representative of all the clinicians who are out there (U08) |  |
|  | Enthusiasm waned over time | So one thing I would mention is that I felt as the project got further and further away from its inception and further away from that initial warm fuzzy buy-in period where I felt like I’ve teethed in the senior leadership team (R01)  So keeping everyone engaged and on board when the timeline of this particular type of research, getting it funded and getting it happening is very slow; it was a barrier (R04)  When you’re doing the initiation there’s a lot of hope I think and there’s a lot of things that you have to learn about each other. And so in that I would say one of the barriers is you can lose people if they don’t, if they don’t really understand that the project is about. Or if they feel like they don’t have input into the project (R05) | Difficult around being able to sustain their interest, their capacity to participate (U08) |  |
|  | Competing priorities made for limited involvement from Research Users | I was trying to engage with the group of people who had a really heavy responsibility load, they’re kind of looking after this, you know these giant [research user organization], they have really big fish to fry and this little you know [product] thing was interesting, innovative, could help them with their mission but probably represented less than one percent of you know what they had on their you know global agenda for the organization. So I feel a little bit of it was that I was competing with many, many other interests on the part of the particular knowledge users (R01)  it doesn’t matter you know how much I would like to be able to take off their plate [partners], they have to do it. (R02)  So they’ve been quite good about that but you know sometimes it’s a little bit like herding cats but that’s because people are busy you know and it’s not that they don’t care, it’s just they got very full schedules and they’re trying to make things work and they’ve got lots of competing you know demands on their time. (R03)  Create a shared understanding of what this project is about and everybody has a little bit different interest (R05)  So some of the difficulties is that in doing that, as a researcher you always have a timeline if you’re applying for funding. And so sometimes it, these people are super busy, have a lot on the go, everybody does but you need to fit in their schedule and that sometimes takes a while. So you have to be super flexible in when you’re willing to meet with people, if it’s at the end of the day, the beginning of the day that they’re willing to meet with you (R06) | Research may not be a priority unless change is possible (U01)  There was at the same time an inter-professional collaboration project going on that was trying to utilize a lot of the resources and they had their own agenda (U03)  So don’t invite me to a meeting if it’s a topic I really don’t care about. (U04)  There’s you know real interest in seeing if we can find evidence around you know looking at longer term impacts on healthcare utilization. And that requires like a you know a randomized control trial that, the recruitment for that takes quite a long time. And then there needs to be a sufficient duration of data collection as well. Of course need a control group. So this, there is a study underway right now but just the timeline is so long to get that kind of a big study finished and you know meanwhile our windows of opportunity sometimes when we’re looking at you know questions around whether to decide to fund something sometimes our timelines are much shorter (U05)  Trying to engage folks who are in the community and less attached to an academic centre is, has been more difficult because they don’t have the same flexibility in terms of participating and being available to be for meetings; similarly non-physician clinicians, sometimes it’s a little bit more tricky because for example, if we think about nurses, again they have less flexibility about the scope of their activities and having time specifically for quality and research orientated activities. So that’s been a little bit tricky around trying to create either formal or informal ways of gathering participants or potential participants to be involved at the table (U08) | And also see and this is one thing that I think is important is an openness to having those relationships. Not all policy-makers are not all service providers or have enough time; a) or b) interested in actually working with researchers necessarily (C02) |
|  | Administrative paperwork for studies is time consuming, need a dedicated person for larger networks | in my lab…there’s probably patients at three hospitals; we might contribute I don’t know, five or six cases over a ten year period. But those five cases, six data-sharing agreements, subcontracts, material transfer agreements, it’ll take eight months to get all those signed and that’s, it’s only gonna be eight months because I’m good at it. It’s every lab across the country that has to do this, to put together a hundred cases or you know a hundred and twenty cases…we’ve created these layers of necessary agreements that are now really onerous to maintain. I had in one of the great glories of life, I have an appointment at both [Hospital] and the [Larger Hospital Network]; I have data-sharing agreements with myself. (R02)  I think I find now that I’m part of other groups but again they’re a larger network like networks where they actually have a dedicated person who can, you know they have a manager of the network. So I find that these groups are much better organized and have much more ability to document everything. You know either recording the meetings or having particular like very extensive minutes or even technical reports (R10) | you have to think broadly about how you can achieve the goal, the overall goal that you have while minimizing the impact on their time and making sure that their time is used wisely and that they feel valued for their time (U08) |  |
|  | Personality of the researcher or research user: Researcher too passionate, not networked, interpersonal dynamics, not open to change, not communicative | And because I was driving it and because I was enthusiastic and because I was passionate about it, they, I feel like they just kind of let me go with it and kind of just diverted their attention elsewhere because it was needed elsewhere (R01)  They [researchers] have the interpersonal skills, they know how to reach out to people, they prioritize the work with the partners; often that, you know their own expense you know of staying up late and doing extra work or you know being delayed on other things. But there are many researchers in my experience who do not have these skills (R05) | I’ve seen really effective professors who just are great at developing networks and those that are not and you know it feels a little inequitable that because of you know personality or you know introversion versus extroversion that you’re, you should not have that network that your colleagues does because they are more inclined to be you know meeting new people and expanding their network (U01)  Sometimes people just don’t get along. And I don’t know how you quantify that in knowledge translation; like you know I might just not like the person and as much as my frontal lobes says, they’ve got valuable work and I really should be listening, the, all the subconscious biases that might be clouding that might not allow me to engage with the person. (U01)  You do need people who are tenured to be, because you know if you’re not tenured you’re in a bit of a vulnerable place and there’s some riskiness to this kind of work because it does take you away from what the usual incentives are in a university environment in terms of your individual research agenda and publications and conference presentation (U07)  Sometimes it doesn’t work. Sometimes you have to actually say to a particular person whose being you know disruptive or toxic in an environment, thank you very much but this isn’t working and you know. I don’t know what the best word be, to fire them? (U08)  Sometimes we’ve encountered where we’ve lost communication with the lead researcher, not knowing what stage there at and then at the end of the research they’re saying, thanks, great. You said you’d host a webinar for us, like oh weren’t you know where has this been and where has this led and it was different then where the original out-set was. So that hasn’t happened often and that was some time ago but certainly that’s a bit of a barrier too (U09)  The structural pieces help and they’re kind of guide posts for partnerships but I really do feel like the other organizations culture and then the individual that you’re working with directly that you’re paired up with at whatever level you’re at. You can be at project-level, you can be at sponsor-level, you could be at executive-level; to me it comes down to the relationship that you build with those people, more so than some of the structural or agreement type of pieces (U10) | that openness from the researcher to change and to really adjust the things that they’re doing in order to or reflect what they’re partners are saying and doing. And researchers, not all researchers are necessarily good at doing that or have thought that that’s the way they should be doing it because they’re kind of trained in opposite way, right? (C02) |
|  | Lack of understanding of the research cycle, research culture | Their [researcher users] timeline and expectation of knowledge is much shorter than the realities of clinical research…So you know they often need to make decisions quickly with kind of knowledge that’s right in front of them. And so I feel like the project like this where you’re asking them to participate in a clinical trial which might take you know a year or two to plan and another year or two to recruit enough patients and then six months after that to analyze the results and then publish data in a peer-review journal. I always get the sense that you know the knowledge users expectations and needs for knowledge is much shorter than our kind of practical limitations for clinical research. So I don’t really know you know what the solution to that is but it’s just something that I have noticed sometimes (R01)  Team were not researchers. So they might not have had the kind of comfort or confidence to start picking away at a scientific protocol and making suggestions and changes (R01)  I think for me is the difficulty in accessing the higher level healthcare administrators. I think it’s difficult. You have to kind of almost know somebody in the field. People obviously they talk to you on the phone but then they try to kind of refer you to somebody else. So I think that’s a bit hard, this kind of barrier in terms of I know what needs to improve on the ground but then often it’s required for a grant application that you actually have knowledge users that are in the higher you know administrative field (R10)  I think I made an assumption that people would know what IKT was but I think we found from the second project that I’m not sure that was the case; that everybody kind of thought they knew what it was but when we talked to some of the knowledge users both in the individual projects that was part of the study as well as our own knowledge user on our team. That I wasn’t sure that they, we were as clear as we could have been. And so that’s a bit of a dilemma because you think maybe people know (R06) | I mean policy-makers don’t really understand the research process and the specificity to which questions need to be posed and methods need to be developed and researchers don’t understand the, I mean this is you know over generalization but typically don’t understand the time pressures and demands on a bureaucrats time. (U01)  And there’s the legislative framework that exists. You might have great model of care that has PSW’s doing something but maybe that doesn’t fit with you know how those health professionals are regulated and they can’t actually do that in practice outside of a research setting. So you know as that, UK science advisor said, law and economics are top dogs and if you don’t address those then you’re probably gonna have a tough time initiating relationships (U01)  You know people like me that don’t know anything about this [initiating partnerships] come up to researchers and go, blah, blah, blah, blah what do you think, right? And then you know the response that you get is either oh that’s really cool, I think I can help you or no. (U02)  On the [Regional] Health side, I think there was a need for and there were people who are willing to be again, build relationships, be flexible and understand that university doesn’t operate the way the health authority does. And there’s different drivers and different incentives in the university environment than there are in the health authority environment. And so it’s a different culture and you have to figure out how to bridge that culture. And we had some people in some key leadership positions that got that and we’re willing to learn to understand that or seek to understand that and yah so (U07) |  |
|  | Geography/  Distance | My capacity to do on the ground really good community engaged work in partnerships is non-existent in communities in [other provinces] where a lot of the programs were at the time. So I needed to draw on my academic partners who were situated in various cities to engage them in the research so that they could do community-based work with [their local] communities…And then you know I would say provincially we did a good job in some provinces but not in others in terms of engaging the provincial partners (R05)  Geographic disbursement. I think humans are still, we’re still wired for you know eye contact and body language and fulsome communication. So I think that working by phone or by skype link, tele link, whatever but through a computer is still challenging for all of us (R07) | So because we’re a smaller geographic health authority we’ve worked, we didn’t have a lot of bench strength at the time of in the organization to build a research endeavour. So we felt that what we needed to do was rather than duplicate and have research institutes within our organization; how could we work with the university that covered the same geographic areas that we did to together leverage the strengths of both organizations (U07) |  |
|  | Misaligned goals and expectations, applying for a grant without clearly defining participation, | And typically at the beginning in initiation phase you have everybody excited there, they’re involved, they want to be involved. But if you don’t have a clear plan for how that involvement is gonna look and people don’t know they’re role, what their roles are and this is where it’s often very difficult because they’re looking to us as the researcher often to be the leader yet we want to co-lead, right? (R05)  But it probably would have been worth to say, alright, just all on the same page here; this is what we think your role is on the study and is that something that you’re prepared to take on? Making it more explicit than what we did. And thinking right at the beginning where would be a home for this particular study (R06)  And sort of expectation management I guess and role clarity. So try and understand what it is everybody wants to contribute to the team and how we’re gonna do it. So I think if there’s lack of clarity around that and sort of if expectations aren’t sort of put out on the table and they’re maligned or misaligned then those things cause barriers, create barriers (R09)  And people who are just trying to put in a PHSI grant or they’re trying to put in a different type of you know project grant; in the end you try to get something that’s functional and that where people are interested in participating. But in whom the full sledged participation may not have been yet optimized and would probably be once the funding has been secured (R10) | To be honest, my ultimate project and goal that I wished has not happened…And partly that was because of everybody else’s priorities and The politically correct way of saying it, is that they decided to focus on what was accomplishable versus what was desirable (U03)  I think sometimes there can just be a challenge in terms of those sectors having different kinds of priorities in a way or different timelines as well. Often times for academic work and you know to for example, to develop certain kinds of evidence requires maybe a certain methodological approach that’s gonna be quite resource intensive and may have quite a long timelines (U05)  I think it would be very helpful though if there was a bit more upfront discussion on what expectations are. I kind of feel like sometimes we have to kind of make it up. It’s like so, here’s where we think we could help but we actually don’t necessarily know enough about your project. (U06)  I think one of the things around any of the partnerships is being very clear and crisp on the expectations. So what’s everybody’s role, like a really clear terms of reference about what’s everybody’s role, what’s the time commitment, what do we expect from folks, what can they expect from us, what’s the reinforcement or what are the rewards of doing it; so that people are coming in with very clear expectations and know what they’re getting themselves in for (U08)  And the more details at a researcher or an investigator or assistant can tell us about the process or what they need from us or the expectation and the timelines the better… But then you know we there’s sometimes that we have to actually say no because we want to make sure that our contribution is both meaningful and that what we’re investing our time and our precious and limited time into or resources to help support dissemination, it actually is aligned with our priorities especially when you know a couple years down the road when the results come out (U09)  Structural things like contracts and procurement and like directives that we have to comply with; policies and procedures within organizations that don’t match up (U10) | Alignment and when you get into formal partnerships; if the end game aren’t aligned it can be really problematic because you’re, you have different accountabilities (C01)  sometimes it is a person that is just not like super interested or doesn’t have enough time to do that (C02) |
|  | Shared forum, repository or space where KUs would share research priorities with researchers | I think a way for researchers to interact with knowledge users or to at least be able to identify who’s who within their particular fields would be useful. I don’t know if that’s practical because it’s, there’s so much variability in everyone’s research field and you know variability and a few of the knowledge users are that might not be practical. But if there were some kind of occasional events or databases along the lines of what I just spoke about but some way of bringing together researchers and policy-makers to talk about different knowledge gaps, knowledge needs and opportunities for working together. That might help a lot (R01)  But for there to be a shared place where the types of questions that are important or the type of innovative opportunities are available are kind of more visible…So if there was a way for policy-makers and knowledge users to be able to kind of post or present their knowledge needs then there might be more pick up from researchers who want to try and fill that knowledge gap (R01)  [Network] really bringing together change agents, innovators, researchers, healthcare administrators; we in particular, topic areas for example, [specific areas] healthcare system. And so those were made a, they’re automatically a bit of a knowledge translation vehicle and a way to both seek collaboratives for a grant or people who are gonna help the success of any knowledge translation products that created to that grant. They’re the kind of people who are going to help to implement or move that forward. Not sure if that structure exists elsewhere in the country but it’s based out of similar work I think from the U.K. and Australia and other places (R07) |  |  |
| What strategies or interventions or tools would support partnership initiation? | Funding for non-research related activities to encourage buy in or participation from research users | We’ve ended up using some of our research money for food because you cannot reward frontline clinicians with time they’re giving to your project. And if you’re bringing them together to do a piece of work in a room face-to-face we found being able to provide food, so you know muffins, coffee, nothing extravagant, pizza…[or other activities such as] data visualization (R07) | So I’ve been talking to the [National Specific Health Professionals Association] you know partners to buy into this. So one of the incentive is that we’ve been batting around would be you know a free conference for somebody [to get involved in project] (U02) |  |
|  | A document or toolbox describing interests, knowledge gaps, a how to start IKT partnerships both formal and informal, ; a written checklist of practical items to pay attention to when starting different types of partnerships | So I think doing your homework, it’s an important strategy and allowing enough time and making sure that you’re not missing anybody. Because in inevitably you will and so what strategies will you use? So some people might say something like, well you know it would be great if there was some sort of clear document that you have that you would describe what your role is as a researcher and not just assume that it’s understood (R06)  May be some kind of a list or a documents that warns the researchers, okay whose never been involved in such a thing of what, you know what they need to pay attention too and you know intellectual property, patent, all these things. People may not think of this as they’re doing, as they’re writing the grants and stuff like that (R10) | Something that would be pretty amazing would be to have somebody write a document that says, so you think that you need to do research in any given area that you worked in. Here’s some of the questions that you need to ask, right? As opposed to you know you, you know an idiot’s guide to how to get research done (U02)  I’m saying, before we go off and say we’re going to partner with everybody we’d better know partnering on what? What’s the level of engagement? And how are we gonna provide feedback? (U04)  No, no one has ever sent anything on roles and responsibilities. nothing on that you know the whole idea of let’s talk first about who does what, when, how to the point of like location. How do the users get noted on publications such, none of that’s ever occurred. But…find rather interesting since the groups that we primarily work with are all heavily, at least for myself are all heavily involved in knowledge translation…not a translation scientist and practitioners and researchers. So knowing how we could be involved and potentially best practices around involving; I don’t necessarily see a lot of that actually to formally being followed through in being approached and working with them. It’s much more friendly, ad hoc you know kind of idea (U06)  there’s other things out there that do talk about having those discussions up front is what are the responsibilities for each of the groups? What are the expectations? How do you handle publications? So that’s at the end but having those discussions at the beginning just helps to set-up that positive trusting relationship (U06)  research that is being done on knowledge exchange, integrated knowledge exchange and what we’re learning about those processes have started to produce some models and some ways of approaching it; that first of all, give validity to the endeavour and also point to some of the things that need to be put in place to, can make integrated knowledge translation a reality… you know checklists and processes are all useful and they can help people get in off into the right direction (U07)  the role clarity and just understanding the roles and the different players and partners. You know we use charters a lot so improvement charters and you know a synthesis of what you know a little bit more than an abstract but a synthesis of what the intent of the research is, is usually really helpful…that shared value and also some shared understanding of what’s required from each. So it’s usually a discussion but I wouldn’t say it’s written in stone but there probably are some you know a checklist kind of a piece might be helpful so everybody’s clear who’s on first (U09) | Toolbox we would have many tools going from organic I would say to very formal. So a lot of formal partnerships are with kind of key stakeholders in the province that you know we would do through contracts and grants and MOU. And in our toolbox we also have the ability to initiate partnerships through different mechanisms like alliances and networks and a bit more informal but can be equally as powerful in terms of the partnership continuum (C01) |
|  | Spend time getting to know your team | So I and I don’t know why maybe well in my past life before I was in academic I was a knowledge broker and that was my entire for years and that was actually my entire focus was building relationship between different, like between decision-makers, clinicians, researchers and so on. So I guess that’s why I focus on it so much. I say that, I don’t have, I don’t know if that’s a strategy or not but sort of I spend a lot time like face-to-face, a lot of meetings, a lot of sort of casual conversation, a lot of like how’s your kids? Like I actually try to get to know people which might sound really funny in a work context but it’s, it’s sort of all-around relationship building (R09) |  |  |
|  | A model based on literature |  | Research that is being done on knowledge exchange, integrated knowledge exchange and what we’re learning about those processes have started to produce some models and some ways of approaching it; that first of all, give validity to the endeavour and also point to some of the things that need to be put in place to, can make integrated knowledge translation a reality (U07) |  |
|  | Funding for partnerships |  | There needs to be resourcing available both in terms of funding for a project perhaps but also in terms of availability of people’s time (U05) |  |
|  | Shared data agreements within the province |  | Like if we had in the province like you know master data-sharing agreements and master privacy clauses for contracts and a standard contract template for participation agreement; like we’ve done that in the clinical trials world quite a bit but we haven’t done that in the partnership world (U10) |  |
